# Communication Skills Training Using Simulation for Integrating Adverse Childhood Experiences into Adult Primary Care Visits for Students

**DOI:** 10.15766/mep_2374-8265.11648

**Published:** 2026-11-03

**Authors:** Martina Jelley, Frances Wen, Julie Miller-Cribbs, Juliana Meireles, Kristin Foulks, Ginger Sutton, Sarah Beth Bell, Michealyn Wyper

**Affiliations:** 1 Professor, Department of Internal Medicine, University of Oklahoma School of Community Medicine; 2 Professor, Department of Family Medicine, University of Oklahoma School of Community Medicine; 3 Professor, Anne and Henry Zarrow School of Social Work, University of Oklahoma; 4 Staff Scientist, Department of Family Medicine, University of Oklahoma; 5 Director of Simulation, University of Oklahoma School of Community Medicine; 6 Senior Grant Coordinator, Office for Research Development and Scholarly Activity, University of Oklahoma School of Community Medicine; 7 Staff Scientist, Office for Research Development and Scholarly Activity, University of Oklahoma School of Community Medicine; 8 Research Coordinator, Department of Internal Medicine, University of Oklahoma School of Community Medicine

**Keywords:** Trauma-Informed Care, Adverse Childhood Experiences, Simulation, Primary Care, Interprofessional, Competency-Based Medical Education

## Abstract

**Introduction:**

Adverse childhood experiences (ACEs) can affect health and well-being throughout the lifespan. Trauma-informed care (TIC) integrates trauma understanding and resists retraumatization through fostering a safe and healing environment. This 3-hour workshop taught trainees competency-based TIC skills to identify potential signs of ACEs and other trauma in their adult patients and sensitively incorporate the impact of these experiences into their care.

**Methods:**

After completing prereadings, third-year medical students (MS3) and second-year physician assistant students (PA2) attended a lecture about TIC and observed a demonstration of a patient interaction. Students then completed a simulated longitudinal outpatient visit with a simulated patient (SP). The four cases of adult primary care patients presented with (1) depression and knee pain, (2) medically unexplained symptoms, (3) chronic back pain, and (4) substance use issues resulting in chronic disease. No SPs initially disclosed trauma, but all had significant ACEs. A faculty member observed the simulation, giving immediate feedback on performance. Workshops were evaluated by students in pre- and postsurveys measuring self-assessed knowledge about TIC, confidence applying TIC principles, and perceived importance of TIC.

**Results:**

One hundred and sixty-seven students completed this training, with 92 completing both pre- and postsurveys. *T*-test analyses indicated significant improvements across self-reported knowledge, importance, and confidence following the training, with the largest increase in confidence in trauma inquiry (+1.06 on a 1-4 scale).

**Discussion:**

This simulation training enhanced students’ understanding of ACEs’ influence on adult health outcomes and strengthened perceived ability to apply TIC principles, including incorporating trauma inquiry into longitudinal care.

## Educational Objectives

By the end of the activity, learners will be able to:

1. Improve knowledge and perception of the importance of ACEs science and trauma-informed care.
2. Increase confidence in the practice of trauma-informed care and trauma inquiry with adult primary care patients.
3. Advance confidence in explaining how trauma can relate to chronic health problems in adult primary care patients.
4. Increase confidence in responding to a patient disclosure of a traumatic past with a trauma-informed approach.
5. Improve confidence in incorporating discussion of resilience and offering resources to a patient after trauma discussion.

## Introduction

Adverse childhood experiences (ACEs) are widely recognized for their profound impact on health outcomes. ACEs refer to traumatic events occurring before age 18, including abuse, neglect, and household dysfunction.^1^ These experiences can lead to toxic stress, which disrupts brain development and other systems, increasing the risk of long-term health issues such as heart disease, diabetes, and mental health disorders. Approximately 63% of U.S. adults have experienced ACEs,^2^ resulting in an annual economic burden of $14.1 trillion.^3^

Trauma-informed care (TIC) is an approach that acknowledges the widespread impact of trauma and seeks to avoid retraumatization while promoting healing and resilience.^4^ TIC involves understanding the effects of toxic stress and integrating this knowledge into clinical practice to provide better support and care for patients with a history of trauma. The correlation between multiple ACEs and adverse adult health outcomes underscores the need for integrating TIC into medical practice. Notably, individuals with four or more ACEs are 3.5 times more likely to feel misunderstood by professionals compared to those without ACEs.^5^

Integrating TIC into clinical practice is associated with an increased perception of partnership between patients and physicians,^6,7^ which may improve patient engagement and health outcomes. Training for health care professionals can enhance their comprehension and application of a TIC framework.^8^ Specifically, embedding these programs within education for physicians and physician assistants (PAs) equips students with a crucial foundation for future trauma-informed clinical practice. The American Medical Association recognizes the importance of physician training in TIC and specifically supports its inclusion in undergraduate medical education (UME) and graduate medical education (GME),^9^ and the Association of American Medical Colleges lists “additional” UME TIC competencies.^10^ Correspondingly, there has been a recent call for the integration of ACEs and TIC training into PA curricula.^11,12^

A growing body of literature has examined the implementation and impact of TIC training in medical education.^11-17^ Prior work describes educational approaches designed to teach core TIC principles, including recognizing and responding to trauma to improve patient engagement and health outcomes.^13^ Reported outcomes include high levels of student satisfaction along with meaningful gains in confidence and perceived competence following TIC training.^13^ Other educational models incorporating trauma-informed communication and experiential learning have demonstrated significant increases in students’ comfort with initiating trauma-related discussions.^11^ Additionally, evidence suggests that even brief educational exposures, such as a single lecture, can significantly improve students’ knowledge and awareness of TIC concepts.^14,17^

Despite significant advancements in integrating TIC and ACEs education into medical curricula, critical gaps remain.^18^ Trainees must acquire the skills to introduce and manage discussions about trauma sensitively and effectively. Simulation-based training can address this deficiency by providing a controlled, safe environment for novice clinicians to practice and refine their skills, enabling them to navigate challenging conversations and build confidence.^19^ A notable effort by Lee et al. (2023) involved simulation-based training for acute care settings, providing students with hands-on experience in TIC practices, resulting in greater preparedness and empathy.^15^ However, an area that needs further development is training students in patient-centered trauma inquiry during continuity adult care visits. The ability to integrate trauma inquiry around childhood trauma in the context of a longitudinal relationship with an adult patient is critical to true patient-centered care. Students may not recognize the prevalence of chronic disease related to childhood trauma. Students surveyed at one medical school ranked internal medicine and family medicine among the lowest specialties likely to have a high proportion of patients with one or more ACEs,^20^ whereas these specialties commonly serve patients with a high level of trauma.^21^

Recently, the National Collaborative on Trauma-Informed Health Care Education and Research (TIHCER) released a set of validated TIC competencies for UME.^22^ This comprehensive list of competencies includes understanding the impact of trauma, recognizing symptoms, and integrating knowledge into practice without retraumatizing patients, and provides a roadmap for integrating these competencies into medical curricula, aiming to enhance training and ultimately improve care.^23^ These efforts underscore the importance of equipping future health care professionals with the skills necessary to provide compassionate and effective care to trauma-affected individuals.

Our training program addresses existing gaps in TIC training for medical and PA students by focusing on simulation-based training tailored to adult primary care continuity visits. Simulation allows reproduction of challenging situations in a safe setting for novice clinicians.^24^ Additionally, it incorporates TIC competencies for UME and follows the competency roadmap.^22^ Designed for students in their clinical years, the training leverages their increased experience and provides opportunities for the immediate application of skills. The specific UME TIC competencies we addressed in this training were knowledge for practice, patient care, and interpersonal and communication skills.^25^ Our goal is to equip students with essential TIC background knowledge (with a focus on ACEs), demonstrate effective trauma inquiry discussions, and enable them to practice these new skills in simulations, thereby reinforcing their abilities, boosting confidence, and ultimately improving their performance in practice.

## Methods

### Context

The development of this training evolved over 15 years of research and experience. Our team has trained a wide variety of learners, including medical and allied health professionals; hospice, prison, and social workers; hospital staff; tribal members; teachers; and an array of undergraduate and graduate students from many fields of study. We have refined and adapted our training to fit the needs of the group. We have developed didactic lectures, role plays, videos, simulations, and a variety of experiential learning activities. This article addresses the training developed for medical and PA students. Some of these efforts have been published and presented in multiple outlets.^18,26-28^

The simulation-based ACEs/TIC training sessions were conducted with students from the University of Oklahoma (OU) at both campuses in Tulsa and Oklahoma City. In Tulsa, the training occurred with the third-year medical and second-year PA students during their monthly topic-focused Student Academy, where they collaboratively learn skills. In Oklahoma City, third- and fourth-year medical students were invited to participate in the simulation-based ACEs/TIC training session as pilots for that campus. Medical students on both campuses received a previous lecture on ACEs and TIC in the Clinical Medicine course in their first 2 years. In addition, the Tulsa medical and PA students received an additional didactic on early-life adversity in a prologue week dedicated to social determinants of health. The study was approved by the OU Norman Campus Institutional Review Board.

### Didactics

The training began with prereadings (Appendix A), which the students completed before the session. Students were provided with resources, including an ACEs infographic, a list of sample language and helpful metaphors for introducing the topic of trauma with patients, and video and book recommendations (Appendix A).

The session began with a 30-minute lecture (Appendix B) that reviewed the concept of ACEs and highlighted how these experiences can lead to negative health outcomes in adulthood, including changes in brain development, stress responses, and immune function. The lecture thoroughly explained the Professional ACEs-Informed Training for Health (PATH) model, which aims to enhance clinicians’ knowledge, sensitivity, and communication skills related to ACEs within the context of longitudinal, patient-centered care. The model focuses on four core communication skills: (1) *Asking and Educating*: introducing ACEs and explaining their impact on health; (2) *Listening*: demonstrating empathy and compassionate understanding; (3) *Accepting*: reducing stigma by validating patient's experience; and (4) *Treatment*: collaboratively engaging the patient on treatment planning.^29^ Lastly, the lecture provided examples and explanations of strategies for preventing or mitigating the negative effects of ACEs, such as deep breathing, meditation, yoga, exercise, recommended self-help books, and a strengths-based approach to patient counseling.

We then demonstrated a patient visit portrayed by two faculty members (Appendix C). One faculty member acted as the primary care provider, and the other acted as the patient. Prior to the demonstration, we summarized the case, which included a medical and social history, as well as the current treatment focus. We made sure to use a realistic and relatable case that accurately represented a patient who had experienced ACEs. Throughout the demonstration, we emphasized the impact of ACEs on the patient's physical and mental health, as well as the importance of a trauma-informed approach when treating patients who have experienced trauma. After the demonstration, students had the opportunity to ask questions and share their thoughts on the demonstration.

### Simulation and Faculty Feedback

The simulated patient (SP) encounter was designed to provide students with practical experience in recognizing and responding to ACEs in a clinical setting. For this communication skills simulation, we used our simulation center SPs—lay community members carefully selected and hired to portray a wide range of patients. Each SP's performance is grounded in a standardized case template (Appendices D-G). The template distills the medical background, emotional cues, body language, and key disclosure points the “patient” must deliver, as well as guidance for communication behaviors the learners should demonstrate. SPs attend an in-person training session where the educator reviews the case, models the encounter, and coaches the group through role-play and feedback cycles. SPs rehearse the patient history, refine nonverbal signals, and practice providing structured feedback so that every learner encounter is consistent, authentic, and educationally sound.

We created four SP cases inspired by patients seen in our community-based clinics, likely to be similar to patients in many adult primary care practices, to help students recognize and manage the long-term health consequences of childhood adversity. Each scenario is adapted from real clinic encounters:

• a middle-aged patient struggling with knee pain and recurrent depression linked to early abuse;
• an individual with medically unexplained symptoms who is convinced they have lupus despite repeated negative lab tests;
• a laborer with chronic low-back pain whose history of neglect affects his interactions with medical care; and
• a longtime smoker with chronic obstructive pulmonary disease (COPD) whose traumatic childhood fuels his maladaptive coping mechanisms and ambivalence about self-care.

Prior to the simulation, students were briefed on the cases they would encounter and the simulation process. During the simulation, one student acted as the primary care provider, while another student and a faculty member observed live-feed video from outside the exam room. The encounter consisted of a 10-minute patient visit, followed by a 5-minute in-room feedback session, during which formative feedback was provided to the student. The feedback was provided by the faculty member, SP, and peer who watched the encounter. Faculty and SPs were given example questions to use during the feedback session (Appendices H and I). To ensure exposure to diverse cases and provide observational learning experiences, each student rotated through simulation stations, participating in one case and observing and providing feedback on another.

The training session concluded with a group debriefing session, where students were encouraged to share their thoughts, ask questions, and provide feedback on the training, including what worked well and what could be improved. The agenda (Appendix J) shows a sample timeline of the training. The simulation time varies with the number of students to be trained, because simulation rounds can be added to or removed from the sample agenda. For the sample, which included 24 students, the session took just over 3 hours with six faculty needed for the simulation session, while only two faculty are needed for the didactic and demonstration section.

### Learner Evaluation

An anonymous online pre- and posttraining survey was conducted to assess the impact of the training. The presurvey was conducted immediately prior to the training, while the postsurvey was administered immediately following the training. The survey consisted of a 14-item questionnaire as well as an open-ended question in the postsurvey to gather impressions of the training (Appendix K). We have created a grading rubric that could be used for objective skills assessment (Appendix L).

The survey included three domains: self-assessment of knowledge (items 1-4), perceived importance of TIC to clinical practice (items 5-7), and confidence in knowledge and skills related to ACEs and TIC (items 8-13). Responses were measured using a 4-point Likert scale ranging from 1 to 4. To evaluate learning outcomes within each domain, composite scores were calculated by averaging responses across items within that domain. Individual survey items were also analyzed separately. Because the pre- and postsurveys were anonymous, responses could not be linked at the individual level to conduct paired samples *t*-tests. As a result, independent samples *t*-tests were used to compare mean scores between groups.

## Results

A total of 167 students (98 MS3s and 69 PA2s) participated in the training. Of these, 138 completed the pretraining survey, and 92 responded to the posttraining survey. Reliability was computed for the composite variables of knowledge, importance of TIC, and confidence in TIC and ACEs. All three had strong reliability scores (knowledge: *\ualpha* = 0.87; importance of TIC: *\ualpha* = 0.86; confidence in TIC and ACEs: *\ualpha* = 0.94).

The Figure shows changes in the three learning domains. Table 1 shows the biggest gain was seen in confidence in providing TIC: mean pre = 2.23 (*SD* = 0.63), mean post = 3.20 (*SD* = 0.50), *P* < .001. The size of this effect was large: Cohen's *d =* 1.68. Substantial gains were also seen in knowledge: mean pre = 2.66 (*SD* = 0.54), mean post = 3.36 (*SD* = 0.49), *P* < .001. The size of this effect was also large: Cohen's *d =* 1.35. There was also a significant gain in attitudes toward the importance of TIC: mean pre = 3.29 (*SD* = 0.61), mean post = 3.62 (*SD* = 0.47), *P* < .001. The size of this effect was medium: Cohen's *d =* 0.58. This more moderate change may be because perceived importance was already high in the pre-assessment, as visualized in the Figure.

**Table 1.**
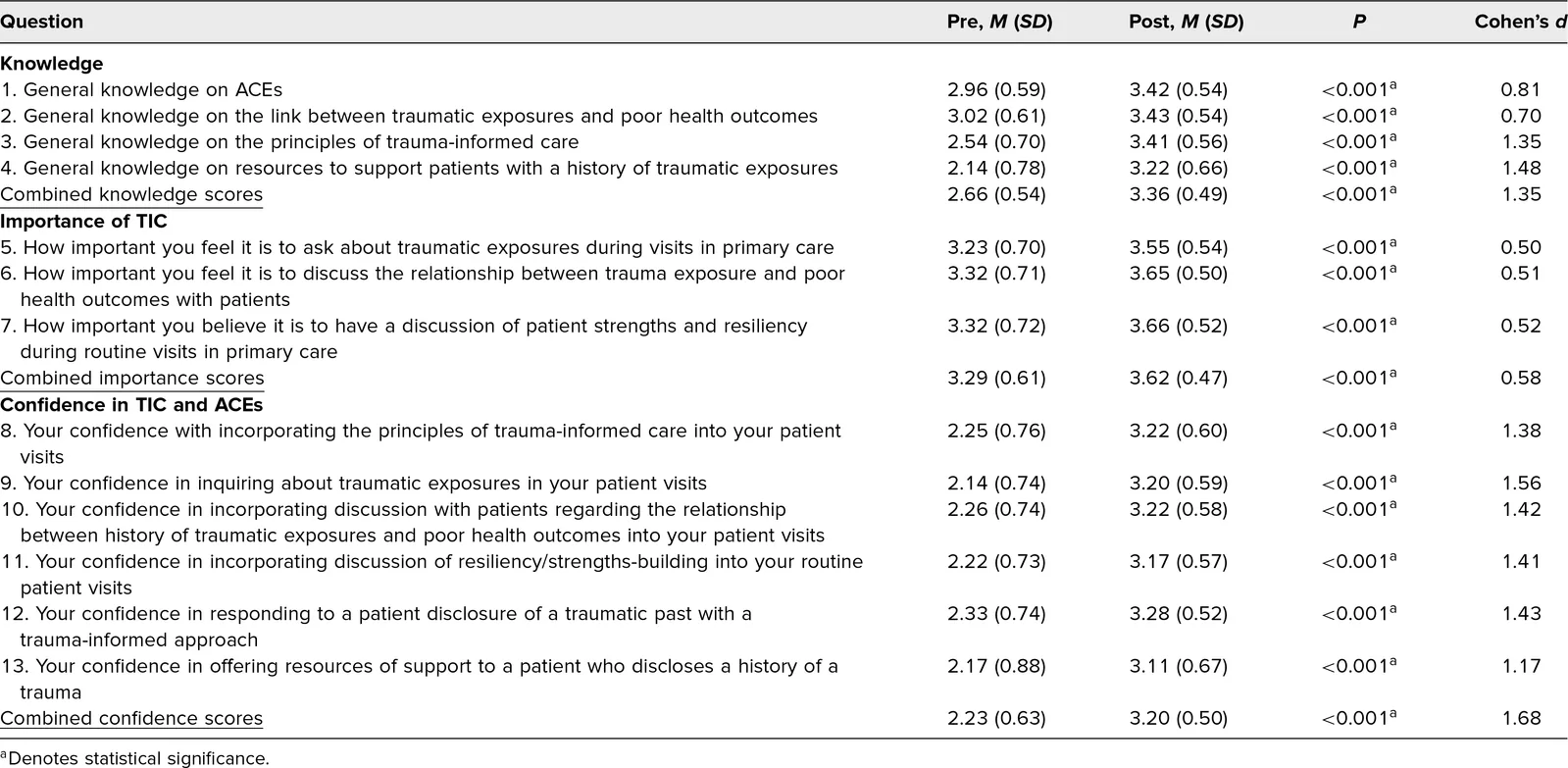
Comparison of Mean Pre- and Postsession Scores for Adverse Childhood Experiences (ACEs) Training

**Figure.**
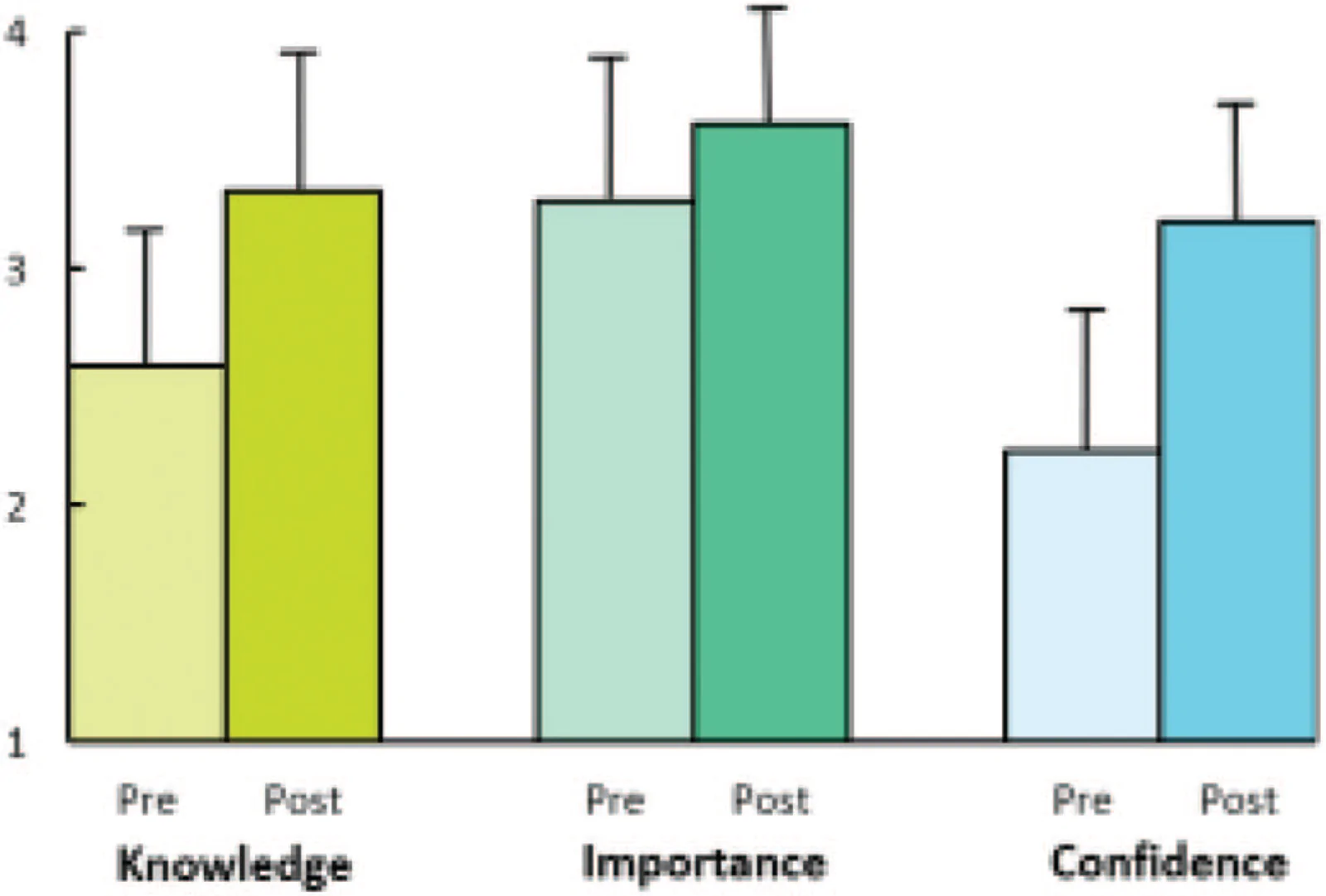
Learning changes in each domain. Error bars denote standard deviations.

Thirty-one students also provided responses to an open-ended question about their experiences with the ACEs training, as shown in Table 2. Of those, 65% (*n* = 17) offered positive feedback, highlighting the value of the case demonstration, the opportunity to engage in a SP encounter, and the immediate faculty feedback as particularly beneficial components. However, 11% (*n* = 3) noted discomfort with being observed by peers during the simulation. Additionally, 24% (*n* = 6) offered constructive suggestions for improving future sessions, such as receiving a sample script for structuring the encounter, distributing case materials in advance, and incorporating more diverse treatment options into the training.

**Table 2.**
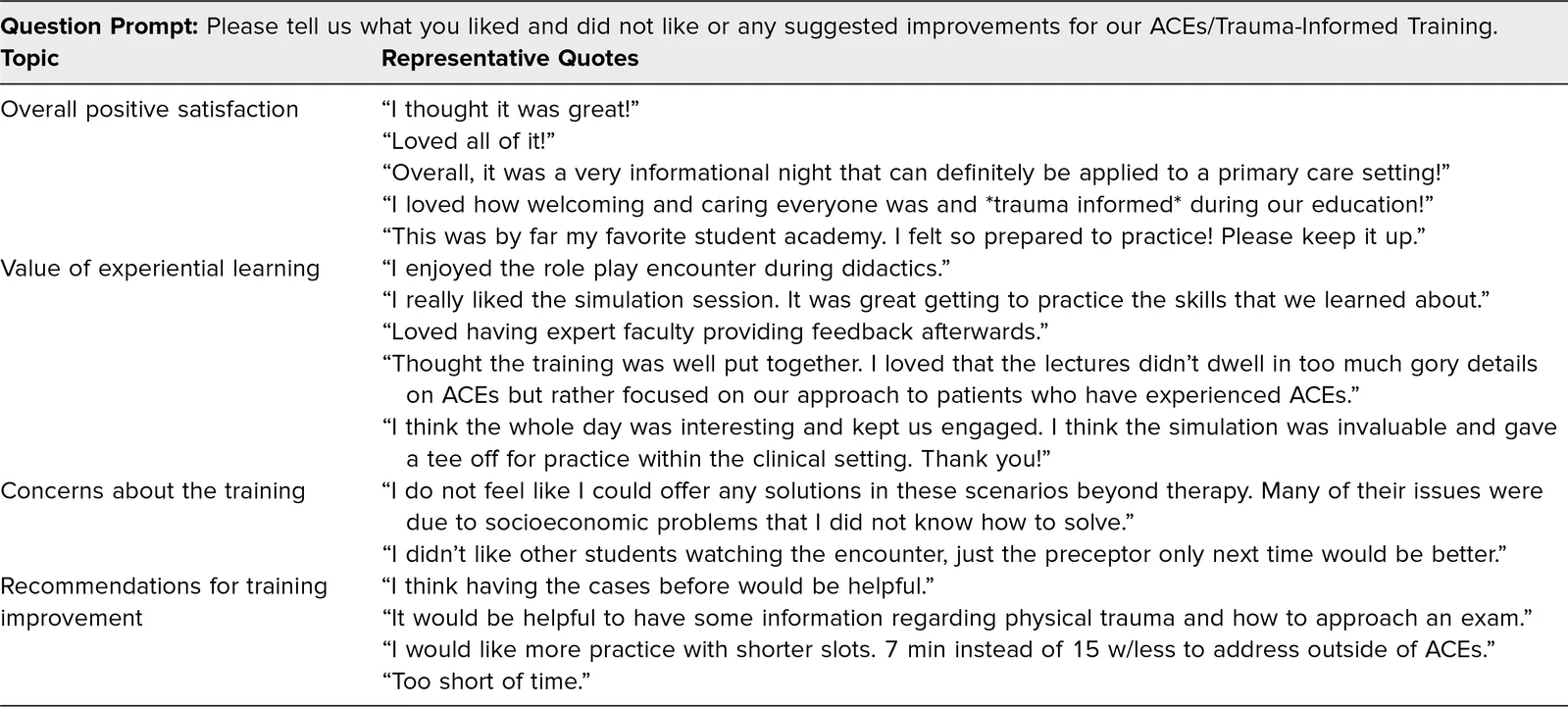
Student Feedback on Adverse Childhood Experiences (ACEs)/Trauma-Informed Care Training

## Discussion

As knowledge of ACEs and their profound influence on adult health continues to grow, there is increasing recognition of the need to prepare future clinicians to address these issues in meaningful and sensitive ways.^12^ In response to this educational gap, we developed a simulation-based training program for medical and PA students that goes beyond knowledge, emphasizing the acquisition and practice of clinical skills. Our training is designed to help students understand the relevance of ACEs in adult medicine and, most critically, to build the communication skills necessary to incorporate this understanding into patient care. The goal is not only to increase awareness of trauma and its effects, but also to prepare students to navigate these complex conversations with competence, confidence, and compassion.

While some existing curricula address ACEs in the context of public health or behavioral science coursework, these are often didactic and rarely offer opportunities for applied skill building. A few programs have incorporated simulations or SP scenarios to teach TIC principles, but these are often limited to pediatrics or mental health contexts, or they target preclinical learners. In contrast, our program is, to our knowledge, the first to use simulation specifically to train medical and PA students to introduce and respond to ACE-related content during adult primary care encounters.

Simulation offers a powerful and unique educational method for this topic. It provides students with a safe, structured environment to practice challenging conversations without risk to real patients. Through realistic, emotionally complex scenarios with trained SPs, students learn to ask about sensitive experiences, respond to disclosures of trauma, and adapt their care approach to align with trauma-informed principles such as safety, trust, collaboration, and empowerment. The use of simulation also allows for immediate feedback and guided reflection, both of which are essential for refining communication skills and deepening students’ understanding of trauma-informed practice.

Another distinguishing feature of our program is its focus on students in their clinical years, rather than in preclinical education. These learners are already engaged in direct patient care and can immediately apply what they learn in real settings. By anchoring the training in realistic adult primary care visits, the simulation emphasizes the everyday relevance of TIC, positioning it not as a specialized skill for behavioral health providers, but as a core competency for all clinicians.

Our training aligns closely with national calls for increased attention to TIC in medical and PA education and supports key competencies in patient-centered care, communication, and social determinants of health. It reinforces the idea that addressing trauma is not separate from medical care, but essential to providing holistic, respectful, and effective treatment. Through simulation, students gain both awareness and practical tools to integrate this understanding into their ongoing practice, ultimately contributing to better outcomes for patients who have experienced adversity.

This study and training initiative are subject to limitations that should be considered when interpreting outcomes and planning for future implementation. The sample was limited to one medical school. A larger and more diverse sample would be needed to draw broader conclusions about the effectiveness of the training across institutions or learner populations. The participant pool included students from both medical and PA programs, which may have contributed to a more diverse student group. Unfortunately, our survey results did not differentiate between types of students or campus, which introduces potential variability in baseline knowledge, clinical experience, and curricular exposure, which could influence how students engage with and benefit from the training. Third, this training required significant resources, including SPs and simulation center time (donated resources for this project), plus a considerable amount of skilled faculty time. Faculty must be trained not only in the content of ACEs and TIC, but also in the effective delivery and debriefing of simulation exercises. The simulation can be adapted to other settings with different resources, as addressed in the Faculty Guide (Appendix H). Additionally, pre- and posttraining survey responses were unmatched, which limits statistical power to detect differences, and response rate for postsurvey was lower than expected despite time allotted to complete. While group-level trends were informative, matched responses would have allowed for a more rigorous evaluation of learning outcomes and program impact. A final limitation is lack of data from an objective measure of skills assessed in this simulation. We have included a grading rubric that could be used for objective skills assessment (Appendix L).

Building on the promising outcomes of this simulation-based training, several next steps are planned to enhance its impact, sustainability, and reach. One key direction is the integration of trauma-informed communication and ACE-related content into core clinical education, particularly within existing clerkships and Objective Structured Clinical Examinations (OSCEs). Embedding these skills into required clinical rotations would reinforce their relevance to real-world practice and signal their importance alongside other essential clinical competencies. Integration into OSCEs would also provide structured opportunities to assess student performance in a standardized, measurable way.

Another critical area of focus is longitudinal follow-up to evaluate the transfer of learning into clinical practice. This could involve direct observation of students during patient encounters, qualitative assessments of how they apply trauma-informed communication skills in real time, or structured follow-up surveys with both students and faculty. Collecting feedback from supervising clinicians could offer valuable insights into how students are using—or struggling to use—these skills in busy clinical settings. Additionally, patient feedback could be incorporated to assess whether the use of trauma-informed approaches is perceived positively by those receiving care.

We also see strong potential for adapting and expanding the training for interprofessional health training, including nurse practitioners, nurses, social workers, and allied health providers. Because TIC is an interdisciplinary priority, developing tailored simulation modules for different roles could foster collaborative, team-based approaches to addressing trauma in health care. Ultimately, future work will focus on evaluating the long-term outcomes of this training on clinical behavior, patient experiences, and system-level practices. By expanding its reach and deepening its integration into clinical education, we hope to contribute meaningfully to a health care workforce that is better prepared to recognize, respond to, and mitigate the impact of trauma in the lives of patients.

## Appendices


ACEs Training Resources.pdfACEs Training Lecture.pptExample for Simulation Demonstration.docxPaula Gardener Case.docxMr. Lewis Case.docxJonathan Jones Case.docxJanet Howard Case.docxFaculty Guide.docxQuick Guide for Postsimulation Feedback.docxACEs Training Agenda.docxPre- and Posttraining Survey.docxClinical Skills Rubric and Checklist.docx
*All appendices are peer reviewed as integral parts of the Original Publication.*

## References

[1] Centers for Disease Control and Prevention (CDC). About the CDC-Kaiser ACE study. CDC. Updated April 6, 2021. Accessed July 17, 2024. https://www.cdc.gov/violenceprevention/aces/about.html

[2] Aslam MV, Swedo E, Niolon PH, Peterson C, Bacon S, Florence C. Adverse childhood experiences among U.S. adults: national and state estimates by adversity type, 2019-2020. Am J Prev Med. 2024;67(1):55-66. 10.1016/j.amepre.2024.02.010 PMC11193602 38369270

[3] Peterson C, Aslam MV, Niolon PH, et al. Economic burden of health conditions associated with adverse childhood experiences among US adults. JAMA Netw Open. 2023;6(12):e2346323. 10.1001/jamanetworkopen.2023.46323 PMC10701608 38055277

[4] Substance Abuse and Mental Health Services Administration (SAMHSA). Trauma-Informed Care in Behavioral Health Services. Treatment Improvement Protocol (TIP) Series 57. HHS Publication No. (SMA) 14-4816. SAMHSA; 2014. Accessed September 17, 2026. https://library.samhsa.gov/product/tip-57-trauma-informed-care-behavioral-health-services/sma14-4816 24901203

[5] Ford K, Hughes K, Cresswell K, Amos R, Bellis MA. Adverse childhood experiences and engagement with healthcare services: findings from a survey of adults in Wales and England. Public Health Wales; 2024. Accessed September 17, 2026. https://phw.nhs.wales/app/uploads/sites/2/2025/08/Adverse-childhood-experiences-and-engagement-with-healthcare-services-Findings-from-a-survey-of-adults-in-Wales-and-England_Report.pdf

[6] Green BL, Saunders PA, Power E, et al. Trauma-informed medical care: patient response to a primary care provider communication training. J Loss Trauma. 2016;21(2):147-159. 10.1080/15325024.2015.1084854 PMC5051697 27721673

[7] Novilla MLB, Bird KT, Hanson CL, et al. U.S. physicians’ training and experience in providing trauma-informed care in clinical settings. Int J Environ Res Public Health. 2024;21(2):232. 10.3390/ijerph21020232 PMC10888540 38397721

[8] Ross DC, Farhat KF, Sayrafizadeh N, et al. A cross-sectional needs assessment for a trauma-informed care curriculum for multidisciplinary healthcare providers. BMC Health Serv Res. 2025;25:426. 10.1186/s12913-025-12568-1 PMC11931758 40128736

[9] American Medical Association (AMA). Adverse childhood experiences and trauma-informed care H-515.952. AMA; modified 2023. Accessed October 13, 2026. https://policysearch.ama-assn.org/policyfinder/detail/Adverse%20Childhood%20Experiences%20and%20Trauma-Informed%20Care%C2%A0%20H-515.952

[10] Association of American Medical Colleges (AAMC). Competency-based medical education (CBME). AAMC. Accessed October 13, 2026. https://www.aamc.org/about-us/mission-areas/medical-education/cbme#other

[11] Brown T, Mehta PK, Berman S, et al. A trauma-informed approach to the medical history: teaching trauma-informed communication skills to first-year medical and dental students. MedEdPORTAL. 2021;17:11160. 10.15766/mep_2374-8265.11160 PMC8180538 34150993

[12] Felter J, Baker A, Lieberman L, Fein J, Reidy MC, Showstark M. Adverse childhood experiences and trauma-informed care. J Physician Assist Educ. 2022;33(3):264-269. 10.1097/JPA.0000000000000449 35917473

[13] Chokshi B, Walsh K, Dooley D, Falusi O, Deyton L, Beers L. Teaching trauma-informed care: a symposium for medical students. MedEdPORTAL. 2020;16:11061. 10.15766/mep_2374-8265.11061 PMC7780743 33409358

[14] Elisseou S, Puranam S, Nandi M. A novel, trauma-informed physical examination curriculum for first-year medical students. MedEdPORTAL. 2019;15:10799. 10.15766/mep_2374-8265.10799 PMC6376894 30800999

[15] Lee CH, Santos CD, Brown T, Ashworth H, Lewis JJ. Trauma-informed care for acute care settings: a novel simulation training for medical students. MedEdPORTAL. 2023;19:11327. 10.15766/mep_2374-8265.11327 PMC10376910 37520013

[16] Onigu-Otite E, Idicula S. Introducing ACEs (adverse childhood experiences) and resilience to first-year medical students. MedEdPORTAL. 2020;16:10964. 10.15766/mep_2374-8265.10964 PMC7499813 32964120

[17] Pletcher BA, O'Connor M, Swift-Taylor ME, DallaPiazza M. Adverse childhood experiences: a case-based workshop introducing medical students to trauma-informed care. MedEdPORTAL. 2019;15:10803. 10.15766/mep_2374-8265.10803 PMC6415008 30931382

[18] Piszczor R, Barry C, Gundacker C, Wallace C, Shibuya J, Perle J. Medical students’ knowledge, attitudes toward, and identification of adverse childhood experiences and trauma-informed care. Perm J. 2024;28(1):91-99. 10.7812/TPP/23.108 PMC10940231 38284149

[19] Wen FK, Miller-Cribbs JE, Coon KA, Jelley MJ, Foulks-Rodriguez KA. A simulation and video-based training program to address adverse childhood experiences. Int J Psychiatry Med. 2017;52(3):255-264. 10.1177/0091217417730289 28893142

[20] Tan J, Dube SR. A pilot study on the awareness and knowledge of adverse childhood experiences science and trauma-informed care among medical school students. Perm J. 2021;25:20.285. 10.7812/TPP/20.285 PMC8784055 35348068

[21] Jelley M, Wen F, Miller-Cribbs J, Coon K, Rodriguez K. Adverse childhood experiences, other psychosocial sources of adversity, and quality of life in vulnerable primary care patients. Perm J. 2020;24:18.277. 10.7812/TPP/18.277 PMC6972546 31905334

[22] Berman S, Brown T, Mizelle C, et al. Roadmap for trauma-informed medical education: introducing an essential competency set. Acad Med. 2023;98(8):882-888. 10.1097/ACM.0000000000005196 36862618

[23] Gerber MR, Jelley M, Potter J. Navigating the roadmap for trauma-informed medical education: application of undergraduate medical education competencies. Perm J. 2024;28(1):169-179. 10.7812/TPP/23.129 PMC10940239 38439660

[24] Leiphrakpam PD, Armijo PR, Are C. Incorporation of simulation in graduate medical education: historical perspectives, current status, and future directions. J Med Educ Curric Dev. 2024;11:23821205241257329. 10.1177/23821205241257329 PMC11131395 38808125

[25] Berman S, Brown T, Gerber M, et al. Trauma-informed care (TIC) competencies for undergraduate medical education. National Collaborative on Trauma-Informed Health Care Education & Research. Accessed September 17, 2026. https://tihcer.weebly.com/uploads/1/2/9/5/129591462/tic_competencies.pdf

[26] Ha M, Rowe A, Hendrix K, et al. Use of metaphor as an explanation tool in adverse childhood experiences simulation training. Perm J. 2023;27(4):72-81. 10.7812/TPP/22.159 PMC10723095 37876251

[27] Miller-Cribbs J, Bragg J, Wen F, et al. An evaluation of a simulation and video-based training program to address adverse childhood experiences. Int J Psychiatry Med. 2020;55(5):366-375. 10.1177/0091217420951064 32883135

[28] Randall K, Miller-Cribbs J, Isaacson M, et al. Using social simulation to teach rehabilitation science students about adverse childhood experiences and trauma informed care. J Allied Health. 2020;49(1):36-44. 32128537

[29] Machtinger EL, Davis KB, Kimberg LS, et al. From treatment to healing: inquiry and response to recent and past trauma in adult health care. Women's Health Issues. 2019;29(2):97-102. 10.1016/j.whi.2018.11.003 30606467

