# Supplementary material for: Communication Skills Training Using Simulation for Integrating Adverse Childhood Experiences into Adult Primary Care Visits for Students
Source: MedEdPORTAL. 2026 Nov 3;22:11648. doi: 10.15766/mep_2374-8265.11648 (PMC13728867; doi:10.15766/mep_2374-8265.11648)
Supplement: Supplementary file 1 [file mep_2374-8265.11648-s001.zip › A. ACEs Training Resources.pdf]

Adverse Childhood Experiences (ACEs) Pre-Reading

Articles:

Machtinger EL, Davis KB, Kimberg LS, et al. From Treatment to Healing: Inquiry and Response to Recent and Past Trauma in Adult Health Care. *Womens Health Issues*. 2019;29(2):97-102. doi:10.1016/j.whi.2018.11.003

Rittenberg E. To Treat My Patient, I Had To Understand Her Trauma. *Health A (Millwood)*. 2020;39(1):161-164. doi:10.1377/hltha .2019.00422

The Truth About Adverse Childhood Experiences (ACEs). *Families Thrive Website*. https://www.familiesthrive.org/blog/2018/4/17/the-truth-about-adverse-childhood-experiences-aces.

Adverse Childhood Experiences (ACEs) Supplemental Resouces

Articles:

Felitti VJ, Anda RF, Nordenberg D, et al. Relationship of childhood abuse and household dysfunction to many of the leading causes of death in adults. The Adverse Childhood Experiences (ACE) Study. *Am J Prev Med*. 1998;14(4):245-258. doi:10.1016/s0749-3797(98)00017-8

Books:

Nakazawa DJ. Childhood Disrupted: How Your Biography Becomes Your Biology, and How You Can Heal. New York, NY: Atria Books; 2015.

Burke Harris N. The Deepest Well: Healing the Long-Term Effects of Childhood Adversity. Houghton Mifflin Harcourt; 2018

van der Kolk B. The Body Keeps the Score: Brain, Mind, and Body in the Healing of Trauma. Penguin Books; 2015.

Schiraldi GR. The Adverse Childhood Experiences Recovery Workbook: Heal the Hidden Wounds from Childhood Affecting Your Adult Mental and Physical Health. Oakland, CA: New Harbinger Publications; 2021.

Metaphors/explanation tools used in ACEs discussions

- <u>The Developing Brain and ACEs</u>
  - Serve and return/Building a brain
  - Brain traffic control
    - Toxic stress and trauma affect one's executive functioning skills (prefrontal cortex) and ability to manage competing life issues. Example is patient with out-of-control diabetes – also has stressful job, family problems, money issues. Unable to manage of all these things at the same time so "a plane crash can happen"
  - Upstairs/downstairs brain
    - Trauma can affect brain development and function causing a person to react with downstairs brain (fight/flight/freeze), rather than thinking brain. Affects executive functioning causing problems with planning and execution of health improvement activities.

- <u>ACEs and Health Consequences- Use of metaphors may help explain the lifetime effects of ACEs</u>
  - Backpack
    - Heavy backpack with too many burdens of trauma
    - Sample language: "Think about carrying a heavy backpack filled with bricks. Those bricks might represent traumatic experiences in your life. You can't see them because they are behind you, but they are weighing you down and not letting you move on with your life and be well. Let's talk about ways that we might be able to help unload some of those bricks from your backpack."
  - Overloaded pick-up truck
    - Similar to backpack. Truck is overloaded with trauma experiences, doesn't run well, possibly breaks down. Need to unload some weight in order to improve health.
  - Tree of trauma
    - Child growing up with trauma, abuse, toxic stress. Soil is unhealthy so branches and leaves show poor outcomes. Good news is that soil can be improved (amended) through watering, fertilization (treatment, improved environment) so that branches and leaves can become stronger.
  - Toxic stress

    - To explain different kinds of stress. Positive, tolerable, and toxic stress.
- Turning up the volume
    - Effects of childhood trauma can “turn up the volume” on symptoms in a patient’s body.
    - Sample language: “Sometimes the toxic stress that our brains and bodies have suffered in the past can change the way our nerve cells work. It can cause nerve signals from our (joints, muscles, etc.) to be amplified when those signals reach the brain. Basically, it’s like turning up the volume on a radio. We want to work together to see if we can help turn that volume back down so that your (pain, other symptoms) is more tolerable.”
- Sunburn
    - A bad sunburn can happen in childhood yet effects of this may nor be seen until years later- skin cancer, wrinkling. Use to explain how something that happened a long time ago can continue to affect a person.

**ACEs** = Adverse Childhood Experiences	**Example Language**
When your patient is struggling, consider "<u>what happened to you?</u>"<br><br>ACEs are well-researched categories of early childhood adversity: abuse, neglect, household problems<br><br>ACEs increase your patient's risk for lifetime consequences for chronic illness, risky health behaviors, poor coping, and unhealthy relationships<br><br>Toxic stress and inflammation are at the foundation of disrupted brain development and some systemic disease<br><br>Consider an ACEs conversation when your patient struggles with trust and engagement, poorly controlled chronic health conditions, adoption of health risk behaviors including substance abuse, repeated/lifetime traumatic experiences<br><br>**Helpful metaphors:** over-loaded truck or backpack, volume control, tree of trauma, air-traffic control<br><br>Adopt an universal trauma-informed approach using accurate empathy, reduction of stigma, health education, collaborative treatment planning	✓ "Hard childhood experiences where you were abused, neglected, or you witnessed violence can affect your adult health and wellbeing. These experiences are called adverse childhood experiences or ACEs."<br>✓ "Do you feel like any of your difficult childhood experiences or other traumas affect your physical or emotional health now?"<br>✓ "I am sorry these things happened to you."<br>✓ "Thank you for sharing your story with me." (If pt chooses to disclose)<br>✓ "Sadly, ACEs are far too common."<br>✓ "Talking about your experiences can help us work together to decide the best treatments for you."<br>✓ "Think about carrying a heavy backpack filled with bricks. The bricks represent traumatic experiences from your childhood. Let's talk about some ways that we might be able to help unload some of those bricks from your backpack."<br>✓ "ACEs turn up the volume on the way our brain processes pain or other difficult experiences. I'd like for us to work together and look at proven ideas that turn the volume down."<br>✓ "The good news is that it's possible to heal from even the deepest wounds of trauma."<br>✓ "Your brain can change."<br>✓ "It's possible to find new, healthier coping strategies for you."
