# Supplementary material for: Communication Skills Training Using Simulation for Integrating Adverse Childhood Experiences into Adult Primary Care Visits for Students
Source: MedEdPORTAL. 2026 Nov 3;22:11648. doi: 10.15766/mep_2374-8265.11648 (PMC13728867; doi:10.15766/mep_2374-8265.11648)
Supplement: Supplementary file 1 [file mep_2374-8265.11648-s001.zip › C. Example for Simulation Demonstration.docx]

**Example for Simulation Demonstration - Debbie & Dr. J Visit**

**Introduction:**
Hello, everyone. We are about to start a demonstration of how a primary care physician in a longitudinal relationship communicates with their patients about their adult patient's history of adverse childhood experiences.
This demonstration will show all of the elements of the PATH model, meaning introducing ACEs, explaining ACEs, demonstrating empathy, engaging in an approach that reduces an individual's sense of stigma, as well as engaging in shared decision making.
Dr. J will be the physician, and I will be the patient. The case that you see today is based on a real patient that I saw in my clinic. This case has similarities to the simulations that you will be engaged in to practice the PATH skills. Remember that the relationship is a longitudinal one. That is our recommendation for where you start in working in this domain of trauma-informed care and more specifically talking about the history of adversity and how it affects a patient's health today.
The other thing to note is that in this demonstration, just as in the simulations that you will be engaged in, all four elements of path will be a focus. However, in real life, this actually plays out over the course of a set of visits, unfolding over weeks or months. All right, so let me tell you a little bit about Debbie. She's 58 years old, divorced female. She's on disability with two adult sons who have substance use disorders. She identifies as female, so she uses the pronouns she and her. She has a history of eight ACEs, including four forms of child abuse and neglect and four types of household dysfunction. Debbie has multiple chronic diseases, including osteoarthritis, major depression, hypertension, and generalized anxiety. She also experiences exertional shortness of breath, which affects her ability to engage in more physical activity and follow up on lifestyle recommendations made by her physician.
Among the health behaviors of focus have included her lack of activity, her poor nutrition, and the lengthy amount of time she spends sleeping and watching TV. Today she comes in to see her physician, Dr. J, for a repeat visit to follow up on knee pain. She also continues to experience a lot of general fatigue and weakness and would like to feel better. Now, the goal for this encounter from a PATH perspective is to explain what ACEs are, and to explain the health effects as they might be experienced by Debbie. In addition, we will focus on maladaptive coping mechanisms, essentially the home remedies that Debbie might be engaged in, which initially in her younger years might have been helpful to deal with stressors, but over time has become maladaptive and might be affecting her health. And the third area of focus will be on pain or increased sensitivity to symptoms. This is meant to take place in a primary care encounter unfolding over the course of 10 to 15 minutes.
So, let's begin.

**BEGINNING OF PATIENT VISIT
Dr. J:**
Hello Debbie. It's good to see you again. How are things going today?

**Debbie:**
Hi, Doctor J.
You know, things are kind of going.
About the same as the last time I saw you. Yeah.

**Dr. J:**
Yeah, I know that we've talked about lots of different things. What seems to be causing the most problems for you lately?

**Debbie:**
Yeah, well, it's my knees. My knees are just killing me.
It keeps me from doing things. I'm not able to stand for very long. And I'm really hoping that you can put in a referral for me to see the sports medicine doctor that you sent me to last time. He gave me some injections into my knees, which
helped me feel a lot better for, you know, at least for a few months. Yeah.

**Dr. J:**
Okay. Yeah. Well, that's definitely something that we can try again and to hopefully get you some relief from your knee pain. I know that that did help at least for a short time, but I feel like it may not help long term, as you probably know, and I think we've had that conversation before?
How's everything else been going with your life and your health these days?

**Debbie:**
Well, um...
You know, my boys are back. I think the last time we met, like maybe one of them was out of the house and, you know, living with some roommates and the roommates were looking at getting him a job at the company that they were working in. Honestly, it's fallen through again. And so he's back in my house. That's my older boy. And he's just basically sleeping in the dining room. My younger son, as you know, has just been with me, he's got the couch.

**Dr. J:**
Wow.

**Debbie:**
Yeah.

**Dr. J:**
That sounds pretty stressful for you.

**Debbie:**
Oh, you bet. You bet. I've been dealing with this their whole lives.

**Dr. J:**
Yeah. I know it’s been hard on you.
So I’ve been thinking about how we can help with your knee pain and help you generally feel better, I wanted to touch on something that we haven't talked about before and see if we can explore a
different pathway. Would that be okay with you to explore something different today?

**Debbie:**
Yeah, yeah, that would be fine.

**Dr. J:**
Okay, so one thing we haven't really talked about is how past experiences can affect your current health, even going as far back as childhood.
And I'll tell you a little bit about why I'd like to know a little bit more about that. We know now that our upbringing, the time that we
spent as young children up until we're adults - the stressors and any kind of difficult times, or traumas that happen to us then can really affect our adult lives and especially our health, including problems with
pain. So can you tell me, what was life like for you when you were a kid?

**Debbie:**
Oh gosh, Doctor J.
You're saying that how my childhood was could be affecting my health now. My childhood was...
It was rough. Yeah, I'll just, one word. Just, yeah, it was rough.

**Dr. J:**

Yeah.
All right. Well, certainly, I'm not surprised because that's actually a very common theme that I've seen with a lot of my patients. And there's a lot of research now that shows how common experiences that have been stressful in childhood are with a lot of people.
What I would like to do is help you understand how that has affected your health. We don't need to know a lot more about what went on. I'm always available to listen if that's something you want to talk about. But really just knowing that you say it was rough tells me that there were probably some events that were so stressful that they caused you what now we commonly know as trauma, even if it wasn't physical trauma.

**Debbie:**
Yeah.

**Dr. J:**
The stress that is going on when you're a kid causes you to have what is known as increased stress hormones in your body. They're kind of like revving up a motor, you know, pushing the gas on a car and revving that up and making your heart beat fast or just making you feel really stressed.
And that's normal when we have a stressful event, but we normally expect that to come back to normal, to kind of a stable idle speed of your body's engine. But what happens when you have stressful events or stressful situations when you're a kid and they stay there all the time, your body never really gets to rest. And so it actually can cause changes in your nervous system, the way your nerves perceive pain, the way they send signals to your brain. And so then in adulthood, it can cause pain to be worse.
It's not something that's all in your head. You're not imagining it. It's just your brain's ability to perceive the pain is heightened, increased, and also your brain's ability to ignore the pain is hampered.
The good news is that brains can change, and there are things that we may be able to work on together to help your brain be able to manage your pain better. I've talked a lot. What do you think about this?

**Debbie:**
Well, you know, at first when you said, you know, stuff in my childhood and how it's affecting me now, I was trying to think back to like when I might have injured my knees or something like that, because that's what I've got, right, knee pain. And my dad was, he was, he was rough with us. And so I was trying to think back. But when you started to talk more about stress and not being able to rest,

**Dr. J:**
Right.

**Debbie:**
Like, that makes sense to me. I, you know.
Yeah.
I really...
I don't know if I ever really felt rest, and...

You know, it's just, there was always something going on, always.

**Dr. J:**
Was it somewhat chaotic when you were a child?

**Debbie:**
Yeah, like I said, my dad was a rough man and he, he drank, drank all the time and he would get into these moods and just
You know.
He would beat us, he would yell scream. I mean, it just was all the things. You know, we...
Um...
It was me, my twin brother, my mom, and, you know, we had to move around a lot. Dad was always losing his job or, you know, it just was.
Yeah, there was always something.

**Dr. J:**
Yeah.
Yeah, that sounds like a lot. There is a lot about the research in this area, that there's a term for that's called toxic stress. Toxic stress is where you have that high level of stress that just kind of never lets up. We all have stress in our lives, but usually it goes up and down. And a lot of times in people and children who grow up in chaotic households with a parent that has a substance use disorder or other issues going on, the stress is always there. And so that definitely causes some changes in the body. And then we're left to deal with it as adults.

**Debbie:**
Ohh.

**Dr. J:**
First, you have to recognize what happened before we can really try to work on it. So I have a few ideas, but we're not going to, you know, finish this conversation in this one setting, but I wanted to just kind of explore with you a few things that
we might be able to start with to work helping to lower your current stress levels, which will help your brain function a little bit better. Would that be okay?

**Debbie:**
Yeah, I think so. I'd like to know more. Yeah. Yeah.

**Dr. J:**
So we know that there is evidence that certain things in our lives that we do can help lower stress levels. We call them stress busters. So there are things such as getting good sleep, eating a healthy diet,
getting some exercise every day, and getting outside with nature, which can actually been shown to be helpful to lower stress levels, even if you're not exercising outside. In Japan, they call it forest bathing, which is, I think, is kind of a fun term.
Things like mindfulness, which some people do meditation, other people just do deep breathing - things to kind of help your brain and your system calm down. And then healthy relationships with friends, with family, and fostering those, those can all help reduce stress. So one thing I was going to ask you about is, because of your knees and what's going on in your home, you might not be able to
get outside much. Do you get outside much?

**Debbie:**
Yeah. No, no, I don't. I live on the 2nd floor and it's really hard to go up and down the stairs A lot. Yeah. Yeah, I, yeah, I'm not able to get out that much. Yeah.

**Dr. J:**
Yeah, so I know it can be difficult for a lot of people because I've talked to a lot of my patients about this, but sometimes even just getting outside, enjoying the breeze, the birds, the trees
If you can get to a park, that’s great, but some people can't do that, so just outside on a patio or a balcony, even if it's only for 10 minutes at a time, that can actually work on the brain to calm the stress that you're feeling.
Is there any place that you think you might be able to go outside for short periods of time?

**Debbie:**
Yeah, my balcony. And I can go listen to the songbirds in the morning before it gets too hot.

**Dr. J:**
Yes. Yeah.

**Debbie:**
Yeah, but so I understand how or I get how this will help my stress. How is that going to help my knee pain?

**Dr. J:**
Yeah, well, we can't promise that it will. But what we do know from lots of research and evidence is when people are able to reduce the stress that's happening in their brain, the pain they feel can go down.

I think sometimes it can be helpful to understand it by using what we know as a metaphor or an analogy. You're old enough to have had a radio that has a volume dial on it, right? So the way we think about it is the noise coming from your knee to your brain has a volume on it. It's either a little bit of pain or a lot of pain. What lowering the stress levels in the brain can do is turn the volume down on that signal that's coming from your knee to your brain. So we hope that not just getting outside, but anything else that you can work on to lower stress levels, and I mentioned some of those things, and we could talk about those more at future visits, all of those may help lower your pain level. Does that make sense? That's just one thing that I wanted to just get started thinking about. In addition to the other things that we'll do, like going back to see the sports medicine doctor, maybe getting an injection, those kinds of things. We can work together to lower your stress levels and this could definitely help the pain. Does that make sense?

**Debbie:**
So.
I'll have less stress and less pain.
Okay.

**Dr. J:**
Yes you have the idea

**Debbie:**
Yeah, I wasn't sure how sitting on the balcony would fix my knee pain, but I get how, it might turn the volume down as you say. Okay, okay.

**Dr. J:**
Yeah, and none of this is, right, none of this is going to be all or none. There are no quick fixes to, but these are just ways that we can think about helping.

**Debbie:**
All right. Well, yeah, I get that. Thank you.

**Dr. J:**
Okay, now.
So I will put in that referral for Dr. C. And in the meantime, if you're able to, can we agree to...
try to have you get outside a little bit more, especially in the morning when it's cool, and just see how that makes you feel.

**Debbie:**
Yeah, I can do that.

**Dr. J:**
That's great. Well, it was good to see you and we'll make an appointment. I'll see you again in a few weeks, okay?

**Debbie:**
Okay, thank you, Dr. J.
