# Supplementary material for: Communication Skills Training Using Simulation for Integrating Adverse Childhood Experiences into Adult Primary Care Visits for Students
Source: MedEdPORTAL. 2026 Nov 3;22:11648. doi: 10.15766/mep_2374-8265.11648 (PMC13728867; doi:10.15766/mep_2374-8265.11648)
Supplement: Supplementary file 1 [file mep_2374-8265.11648-s001.zip › D. Paula Gardener Case.docx]

**DOOR NOTE**

**PATIENT/CLIENT INFORMATION:**

**NAME:** Paula Gardner

**CHIEF CONCERN/REASON FOR VISIT:** Multiple symptoms, wants lupus test

**SETTING:** Ambulatory clinic, scheduled visit

**PERTINENT INFORMATION**

- You are the PCP for this patient and have seen her several times over the past year.
- She has a history of many symptoms that have been hard to diagnose, has seen two rheumatologists and given diagnosis of fibromyalgia, but all serologic tests for other inflammatory diseases have been negative. The symptoms of rashes, mouth ulcers, and fevers have not been seen by clinicians during any of her visits. She also has diagnoses of bladder pain syndrome and chronic fatigue.
- Patient is frustrated that no one can find out what is wrong with her.
- You have not discussed any history of childhood trauma with this patient.

**INSTRUCTIONS:**

Take a focused history and counsel patient on treatment plan. You can address the lupus-like symptoms and patient request for another lupus test briefly but your goal is to do a trauma inquiry with pt and if positive, discuss how that might be affecting her current health. If time permits, you might begin discussing treatment options for patients with history of trauma.

**ATTACHMENTS:**

**Facilitators:**

Print door notes for each room, observer schedule for each station.

Print RWJF ACEs infographic, attach to clipboards.

Print door notes.

**STANDARDIZED PATIENT TRAINING MATERIALS**

| **OVERVIEW** | **Event title:** Chronic disease and ACEs  **Event objectives for learner:**   1. Briefly address pt’s chief concern 2. Perform sensitive trauma inquiry including how pt feels trauma has affected health. Allow pt to determine extent of disclosure 3. Educate pt on how trauma affects the brain and body 4. Make a plan with patient for next steps   **Event format:** Individual, in-person encounter |
| --- | --- |
|  | **Case authors:** M.Jelley, F. Wen, K. Coon, J. Miller-Cribbs, K. Foulks  **Date developed:** 2015  **Date revised:** 3/2023 |
| Affect or physical presentation: | Initially appear frustrated, defensive, anxious, irritable, and guarded. At the beginning of the encounter your body language is closed-off (e.g., cross your arms, look away from learner). Express frustration that your lupus tests keep coming back negative and you keep experiencing these symptoms.  As learner develops rapport with you, you may become less frustrated. See affect guidelines below. |
| Clothing/moulage: | Well-dressed, clean |
| Patient name & pronouns: | Paula Gardner |
| Sex and age range: | Female, 30-40 years |
| Chief complaint: | Continued lupus symptoms, requests another lupus test |
| Chief concern: | Undiagnosed lupus |
| Opening statement: | “I really think I have lupus and want to get tested again. These symptoms keep coming back.” |

**HISTORY OF THE PRESENT ILLNESS (HPI) TIMELINE**

Please refer to questioning & affect guidelines for instructions on delivery of this information.

| **8-10 yrs. ago** | Started having intermittent episodes of skin rashes, mouth ulcers, low grade fevers, joint pain, chronic fatigue. |
| --- | --- |
| **1 year ago** | Last evaluation by rheumatologist who thought she may have fibromyalgia but not lupus or other connective tissue disease. |
| **Since last clinic visit** | Has had continued episodes of symptoms that resolve before she can be seen in clinic. |

**QUESTIONING GUIDELINES**

| Information ***freely given*** if asked open-ended question(s): | **Qs about Reason for Visit:**   1. **Reassess for Lupus:** You still have mouth ulcers, rashes, pain in your joints, chronic fatigue, and occasionally feel feverish with temp of 100F. You know that you’ve been tested before, but you want to get another test for lupus because of your symptoms. You also want a prescription of prednisone for the pain.   **Qs about ACEs and Trauma History:** Your parents got divorced when you were 9. Your mom had a hard time, so you left home when you were 16. You got married when you were 17, but it didn’t work out and your got divorced at 19. You don’t regret the marriage because you have your 15-year-old. daughter.   - If the student asked this open-ended question compassionately, you can also say “My parents divorced because my dad was sexually abusing me.”   **If learner asks if anything has changed since your last visit or current stressors:** “Things have been a little stressful”. Your daughter was diagnosed with ADHD but even though the school was working on an IEP (individualized educational plan) she dropped out of school**.** Your pain from fibromyalgia and bladder pain syndrome has been bad.  **Qs about Efforts on Your Own:** You take your medications as prescribed but the pain medications aren’t helping. You’d like to see if the dose can be increased or another medication tried. You’d like to get tested again for lupus because you are sure that having the right diagnosis will get you the right treatment plan so you can feel better. |
| --- | --- |
| Information provided ***only when learner asks specific question(s)***: | **Qs about Reason for Visit:**   1. **Lupus testing** Previously tested by 2 rheumatologists, but you think your symptoms mean you have been undiagnosed and you want another physician to repeat the test. 2. **If there is a depression screen/evaluation:** You have been feeling unhappy for a long time that you don’t remember what happy is like. You are more anxious, irritable, and down since your daughter quit school and you both are fighting more. You feel guilty over what is happening because it’s like a repeat of your own teenage years. You are having trouble falling and staying asleep, and you’ve lost your appetite, though you haven’t noticed a lot of change in weight. You are low in energy and aren’t doing much except the essentials around the house. Not suicidal/homicidal.   **Qs about ACEs and Trauma History:**   1. **Childhood** – Your mom divorced your dad because of his sexual abuse of you, but she ended up resenting you because of how hard life was as a single parent with struggling to work, pay the bills, and keep food, rent and utilities going. The way your mom treated you was “bad”.    a. **If the student asks compassionately what this was like for you**, “I felt unloved and unwanted. My mom would say she was cursed with me.” 2. **Childhood sexual abuse** –You don’t really think about it because it was so long ago. You have never talked to a counselor or therapist.   **Link Between ACEs and Trauma History and current health or functioning (*this could be a question asking you if you are aware of a link, or it could be the learner providing a statement that there is a link and then using a metaphor or biological explanati*on)**   1. You are initially reluctant to accept the link because you are afraid this will invalidate your pain, what you think is the likely diagnosis of lupus, and you are concerned what this may mean for your diagnosis of fibromyalgia. “I get how my past may make me more depressed and have problems with my kid. But my pain is real, so are my rashes, mouth ulcers, and fevers.”    a. **…if the learner provides another attempt at an explanation** **but it is hard to understand or not delivered in empathic or supportive way “**OK, so you are saying that what happened in the past is affecting my physical health, my pain now. But I’m still not sure how we get from the past to now. I wake up so stiff and sore in the morning. How does what I went through at age 7 cause this?”    b. **…if the learner provides another attempt at an explanation** **and it is delivered in empathic or supportive way and/or is understandable** Express that you can see how there could be a link, and ask “what do I do now?” and then be open to suggestions for your treatment |
| Information that ***must be provided***, even if not elicited: | When asked about reason for visit – lupus testing, say “I think I really need a test for lupus because everything I’m going through adds up to lupus. I’m sure I’ve gone undiagnosed and I really need answers so I can get past this.” |
| Any **challenging questions** that patient could ask: | If the learner attempts a link between your ACEs and trauma with your symptoms that does not make sense to you, say “so you think all of these things that I’m going through are all in my head?” |
| SP response if no attempt to connect ACEs/Trauma to health is provided: | Focus on further testing and evaluation as the solution to your concerns about your undiagnosed symptoms. |

**AFFECT GUIDELINES**

Affect changes throughout encounter based on dependent conversational variables.

| **If learner…** | **…then patient** |
| --- | --- |
| **Demonstrates active listening:** reflection of your concerns, validating feelings and experience, etc.  “I can understand”  “Thank you for telling me all of that”  Sounds like life was difficult for you as a child “It sounds like…”  **No or poor active listening:** does not reflect concerns, validate feelings and experience  Examples:  No verbal/nonverbal expression of empathy, but instead asks another question or directs conversation to another topic | Becomes visually less downcast, less flat in affect. Make better eye contact. Become more open to answering questions. Share the severity of your problems more readily.  Become more downcast and irritable. Cross your arms, hug your body, shake your leg in agitation. Provide brief, unelaborated answers to questions that don’t get to your goal of lupus testing. Insist instead that the doctor focus on your request for more testing. |
| **Partners with patient:**  “What do you think about…”  “Do you have any ideas?”  “How can I help you?”  “What do you want?”  “Let’s solve this together?” | Respond in kind by being willing to problem solve.  “I am really tired of feeling tired, and it’s a pain being in pain. I’d like to feel different”  “Well, I haven’t been to counseling before. How can this help?”  “I’ve been managing on my own for so long. I like trying to solve this together.” |
| **Gives information to patient about ACEs, neurobiological stress, link to physical and mental health conditions in adulthood:**  “Research shows that there is an association between difficult experiences in childhood and health problems in adulthood like depression and chronic pain…”  “ACEs are like a loaded backpack…  “Sometimes stress becomes so much it becomes toxic to us…”  “This treatment can help with…” | Be more physically open and curious about the discussion and trusting of the information provided.  Increase your energy and increase your interest, especially if the student talks about research about ACEs and trauma impacts on health. Ask where you can learn more. |
| **Prescriptive with patient:**  “You need to…”  “I want you to…” | Argue, resist, question, cross arms.  “Yes, but….”  “I’ve tried that and it doesn’t work” |
| **If learner is considerate, shows active listening, and makes an attempt (however successful) at linking ACEs and trauma to current health and functioning:** | Regardless of whether learner provided understandable information about ACEs and adult health outcomes, contribute to the discussion on developing a plan.  You ultimately will agree to treatment for your pain, e.g., PT and continuation of your nonnarcotic pain medications, treatment for depression.  If the learner has been empathic and considerate, you agree to other plans to address family needs, social isolation, e.g., individual and/or family therapy, or identifying/joining social groups. |

**PATIENT’S PAST MEDICAL HISTORY**

| General health: | Good |
| --- | --- |
| Childhood illnesses: | N/A |
| Adult illnesses: | Fibromyalgia, bladder pain syndrome, chronic fatigue |
| Hospitalizations: | None (except childbirth age 18) |
| Surgical procedures: | **Procedure** \| **Location** \| **Date (year)**; Hysterectomy \| N/A \| N/A; Cholecystectomy \| N/A \| N/A |
| Injuries/traumas: | N/A |
| Allergies/drug reactions: | **Allergen** \| **Reaction**; Multiple drug allergies \| Various rashes, fever |
| Current medications: | **Name** \| **Treatment use** \| **OTC/Prescription** \| **Dosage**; Tramadol \| Pain \| Prescription \| 50 mg q6h prn pain; Pregabalin \| Pain \| Prescription \| 75 mg 3x/day; Amitriptyline \| Bladder pain \| Prescription \| 50mg qhs; CBD \| Pain/sleep \| OTC \| |
| Psychiatric history: | Thoughts of suicide: \| No; Thoughts of harming others: \| No; Mental health treatment: \| None |
| Family medical history: | Father who struggled with alcohol abuse most of the patient’s childhood.  All other N/A. |

**SOCIAL HISTORY**

| Patient description | Marital status: \| Divorced; If married, how many years? \| 2; Children: *(Number & ages, if relevant)* \| 1, daughter age 15; Occupation: \| Receptionist – currently on disability |
| --- | --- |
| Lifestyle/behavioral risks:  *(Include amount and duration)* | Eating habits: \| N/A; Exercise: \| N/A; Sleeping habits: \| Poor quality sleep; Stress: \| Family: Daughter struggles with uncontrolled ADHD and has dropped out of school. Health: Undiagnosed, recurrent lupus-like symptoms.; Tobacco: \| Non-smoker; Alcohol: \| Rare social drinker; Street drugs: \| None |
| Childhood trauma history: | - Parents both abused alcohol. - Sexually abused by father at age 8. - Parents divorced at age 9, shortly following revelation that father sexually abused you. Mother blamed you for the divorce for many years. - Mother emotionally abused and neglected you after obtaining custody of you. - Left home at age 16, married at 17, child at 18, divorced at 19. |

GUIDELINES FOR VERBAL FEEDBACK:

- Should be given to the learner from the ***patient perspective!***
- Should always include a behavioral example: “When you said x, it made me feel y.”
- Do not comment on medical information!
