# Supplementary material for: Communication Skills Training Using Simulation for Integrating Adverse Childhood Experiences into Adult Primary Care Visits for Students
Source: MedEdPORTAL. 2026 Nov 3;22:11648. doi: 10.15766/mep_2374-8265.11648 (PMC13728867; doi:10.15766/mep_2374-8265.11648)
Supplement: Supplementary file 1 [file mep_2374-8265.11648-s001.zip › E. Mr. Lewis Case.docx]

**DOOR NOTE**

**PATIENT/CLIENT INFORMATION:**

**NAME:** Mr. Lewis

**CHIEF CONCERN/REASON FOR VISIT:** Worsening shortness of breath

**SETTING:** Ambulatory clinic, scheduled visit

**PERTINENT INFORMATION:**

- You are the PCP for Mr. Lewis and have seen him several times over the last year. He always comes in with his wife who often does the majority of the talking.
- Mr. Lewis has struggled with controlling his chronic disease – HTN, DM2, CKD, COPD. He has not been able to quit smoking or drinking alcohol despite several attempts.
- You have never discussed anything that happened to Mr. Lewis in his childhood.

**INSTRUCTIONS:**

Take a focused history and counsel patient on treatment plan. You can address the ShOB briefly but your goal is to do a trauma inquiry with pt and if positive, discuss how that might be affecting his current health. If time permits, you might begin discussing treatment options for patients with history of trauma.

**ATTACHMENTS:**

**Facilitators:**

Print door notes for each room, observer schedule for each station.

Print RWJF ACEs infographic, attach to clipboards.

Print door notes.

**STANDARDIZED PATIENT TRAINING MATERIALS**

| **OVERVIEW** | **Event title:** Chronic disease and ACEs  **Event objectives for learner:**   1. Briefly address pt’s chief concern 2. Perform sensitive trauma inquiry including how pt feels trauma has affected health. Allow pt to determine extent of disclosure 3. Educate pt on how trauma affects the brain and body 4. Make a plan with patient for next steps   **Event format:** Individual, in-person encounter |
| --- | --- |
|  | **Case author:** M. Jelley, F. Wen, K. Coon, J. Miller-Cribbs, K. Foulks  **Date developed:** 2015  **Date revised:** 2026 |
| Affect or physical presentation: | Slightly anxious, fatigued, lets wife do quite a bit of the talking, especially at first |
| Clothing/moulage: | Casual, generally clean but not fancy dresser. |
| Patient name & pronouns: | Mr. Lewis (he/him) |
| Sex and age range: | Male, 40-70 |
| Chief concern: | Shortness of breath |
| Opening statement: | “I’m having more trouble breathing. I get winded so easily now that it’s making it hard to work. I really need to work, and I’m afraid if this goes on, I’m going to have to retire.” |

**HISTORY OF THE PRESENT ILLNESS (HPI) TIMELINE**

Please refer to questioning & affect guidelines for instructions on delivery of this information.

| **2 yrs. ago** | Started seeing current PCP, comes in approximately every 4-6 months. Had a cough and occasional ShOB at that time. Also had HTN, DM2, and CKD – not at goal. |
| --- | --- |
| **1 yr ago** | Began having more frequent problems with ShOB, began missing some workdays due to breathing issues. |
| **3 mos. ago** | Last visit at clinic, new inhaler prescribed, smoking cessation discussed |
| **Since last clinic visit** | Missing more days of work and most days has ShOB, some days with increased cough. Boss has told him he needs to stop missing so much work. Doesn’t sleep well, can’t sleep at all unless drinks in evening. Tried to cut back on smoking but unable to due to “nerves”. |

**QUESTIONING GUIDELINES**

| Information ***freely given*** if asked open-ended question(s): | **Qs about Reason for Visit:**  **Mr. Lewis: Shortness of Breath (ShOB)** Lately, you have been getting winded more easily and you have to sit down to rest a lot. You work as a meat cutter, and you are taking more breaks to rest, and missing work a lot lately. You’d like to feel better so you can keep working, and are worried that if this continues, you will have to retire and you can’t afford to do that.  **Mrs. Lewis: ShOB** You express your concern about how your husband is doing and that he gets winded after walking even short distances. You describe how he’s such a hard worker and has provided for you all these years. You are concerned about what he’d do in retirement, but he just might have to because he loses his wind so much and is still smoking. You tell the doctor that he misses taking his medications especially when he’s been drinking.  **Qs about ACEs and Trauma History:**  **Mr. Lewis:** Your dad was a moonshiner and went to prison for manslaughter. So as the oldest of 9 kids, at the age of 12 you left school to look after the family. You’ve been smoking and drinking for as long as you can remember because that’s just what you did.  **Mrs. Lewis:** You affirm what your husband says and express support/praise for him for doing what he had to do to make sure his family didn’t starve. “He’s always been a provider.”  **If learner asks if anything has changed since your last visit or current stressors:**  **Mr. Lewis** You can’t manage anymore at work because of getting winded so easily and just being tired. **Mrs. Lewis:** You say that your husband comes home exhausted every night now, but he’s also more winded on weekends when more relaxed, like when just walking around their property.  **Qs about Efforts on Your Own:**  **Mr. Lewis:** You try to not overexert yourself so you can preserve your energy. After work, you come home tired, so you sit in your chair and “relax”.  **Mrs. Lewis:** You tell the doctor that “relaxing means smoking and drinking until he falls asleep.” |
| --- | --- |
| Information provided ***only when learner asks specific question(s)***: | **Qs about:**   1. **Smoking**   **Mr. Lewis** You admit that you keep smoking but it’s so hard to quit because you’ve been smoking your whole life, since you were 11. You know that if you quit smoking you probably wouldn’t have so many issues with your breathing, but you’ve tried before with the gum and patches and it didn’t work. You’ve never tried medications but you “take too many pills now as it is.”  **Mrs. Lewis:** You are encouraging to your husband, “I’m sure that you would feel better, and maybe we can try.”   2. **Drinking**   **Mr. Lewis:** You were in a hospital and tried AA or “something like that” and it just doesn’t work for you because “it’s all people talking about their personal stuff and I’m not one to be like that.”  **Mrs. Lewis:** You confirm that your husband is a private person and there are things he went through that only you know about but even the two of you don’t talk about it.   3. **Medication adherence**   **Mr. Lewis:** You do your best to take your medicines every day but sometimes you forget.  **Mrs.** **Lewis:** “He really forgets after having a few beers at night.”  **Qs about ACEs and Trauma History:**   1. **ACEs** – **Mr. Lewis** – Your response to a specific question on ACEs or past trauma is “that’s all in the past.”    a. **If the learner asks compassionately, Mrs. Lewis should look over at Mr. Lewis and then say “**He also experienced bad treatment from his dad, yelling and whippings. After his dad went to prison, his mother divorced him and he didn’t ever see his father again. He also took on a lot helping to keep food on the table for the family”.   **Link Between ACEs and Trauma History and current health or functioning (*this could be a question asking you if you are aware of a link, or it could be the learner providing a statement that there is a link and then using a metaphor or biological explanation*)**   1. **Mr. Lewis:** Initially express some confusion, “I don’t think these things I experienced as a kid are affecting me now. I was brought up to move on from the past and not dwell on it.”    a. **…if the learner provides another attempt at an explanation** **but it is hard to understand or not delivered in empathic or supportive way… Mr. Lewis: “**So, the things that I went through as a kid affected me back then. But I’m still not sure I get how it affects me in the here and now.”    b. **…if the learner provides another attempt at an explanation** **and it is delivered in empathic or supportive way and/or is understandable …Mr. Lewis** express that you can see how there could be a link, and ask for more explanation. Then ask “Ok, what can we do now” and initially show concern about how difficult it will be to change but ultimately be open to suggestions for your treatment. **Mrs. Lewis**: be supportive and express hope that the treatments could help Mr. Lewis begin to feel better |
| Information that ***must be provided***, even if not elicited: | N/A |
| Any **challenging questions** that patient should ask: | If the learner attempts a link between your ACEs and trauma with your ShOB that does not make sense to you, say “How do you figure what happened back then matters now? The past is the past, you just have to move on, I always say.” |
| SP response if no attempt to connect ACEs/Trauma to health is provided: | Focus on medications or some treatment like a “puffer” that could get help you get your breath back so you can keep working. |

**AFFECT GUIDELINES**

Affect changes throughout encounter based on dependent conversational variables.

| **If learner…** | **…then patient (Applies to Mr. and Mrs. Lewis)** |
| --- | --- |
| **Demonstrates active listening:** reflection of your concerns, validating feelings and experience, etc.  “I can understand”  “Thank you for repeating all of that”  ““It sounds like…”  “Thank you for sharing”  **No or poor active listening:** does not reflect concerns, validate feelings and experience  Examples:  No verbal/nonverbal expression of empathy, but instead asks another question or directs conversation to another topic | Becomes visually more relaxed and comfortable. Make better eye contact. Become more open to answering questions. Share the severity of your problems more readily.  Become more distant. Cross your arms. Make less eye contact. Provide brief, unelaborated answers to questions about your past or current stressors. Redirect back to your concern about your current health (SOB) in the present. |
| **Partners with patient:**  “What do you think about…”  “Do you have any ideas?”  “How can I help you?”  “What do you want?”  “Let’s solve this together?” | Respond in kind by being willing to problem solve.  “Well, I tried before to quit smoking and did for a while. I guess I can try again.”  “I don’t like being winded and not able to get my breath. I can try that.”  “I’ve always looked after things first for my mother and brothers and sisters and then for my wife. I need to keep going for us both. I will try that….” |
| **Gives information to patient about ACEs, neurobiological stress, link to physical and mental health conditions in adulthood:**  “Research shows that there is an association between difficult experiences in childhood and health problems in adulthood like depression and chronic pain…”  “ACEs are like a loaded backpack…  “Sometimes stress becomes so much it becomes toxic to us…”  “This treatment may help with…” | Be more physically open and curious about the discussion and trusting of the information provided. |
| **Prescriptive with patient:**  “You need to…”  “I want you to…” | Argue, resist, question, cross arms.  “Yes, but….”  “I’ve tried that and it doesn’t work.”  “Why would I try that when I know it doesn’t work?”  “Why should I waste my time?” |
| **If learner is considerate, shows active listening, and *makes an attempt* (however successful) at linking ACEs and trauma to current health and functioning:** | Regardless of whether learner provided understandable information about ACEs and adult health outcomes, contribute to the discussion on developing a plan.  You ultimately will agree to treatment for your  COPD, smoking and/or drinking, *e.g.*, smoking cessation, reduction in drinking.  If the learner has been empathic and considerate, you agree to think about counseling and talk it over with Mrs. Lewis. |

**PATIENT’S PAST MEDICAL HISTORY**

| General health: | Fair |
| --- | --- |
| Childhood illnesses: | N/A |
| Adult illnesses: | Chronic obstructive pulmonary disease (COPD), type 2 diabetes (DM2), hypertension (HTN), stage 3 chronic kidney disease (CKD3) |
| Hospitalizations: |  |
| Surgical procedures: | **Procedure** \| **Location** \| **Date (year)**; N/A \| N/A \| N/A |
| Injuries/traumas: | N/A |
| Allergies/drug reactions: | **Allergen** \| **Reaction**; N/A \| N/A |
| Current medications: | **Name** \| **Treatment use** \| **OTC/Prescription** \| **Dosage**; Metformin, \| Diabetes \| Prescription \| 1000mg bid; Amlodipine, lisinopril \| HTN \| Prescription \| 5mg, 20mg; Albuterol inhaler \| COPD \| Prescription \| 2 puffs prn |
| Psychiatric history: | Thoughts of suicide: \| No; Thoughts of harming others: \| No; Mental health treatment: \| None |
| Gynecologic history: | N/A |
| Family medical history: | Father who struggled with alcohol abuse most of the patient’s childhood.  All other N/A. |

**SOCIAL HISTORY**

| Patient description | **Marital status:** \| Married; **If married, how many years?** \| 25; **Children:** ***(Number & ages, if relevant)*** \| 3; **Occupation:** \| Meat cutter |
| --- | --- |
| Lifestyle/behavioral risks:  *(Include amount and duration)* | **Eating habits:** \| N/A; **Exercise:** \| only work related; **Sleeping habits:** \| Hard time falling asleep without alcohol; **Stress:** \| worries he will lose his job; **Tobacco:** \| 2 ppd since age 11; **Alcohol:** \| 4-6 beers every weeknight, more on weekends; **Street drugs:** \| none |
| Childhood trauma history: | - Grew up in rural setting, oldest boy in family of 9 children. - Asked to take on responsibility for providing for family at age 12 when father sent to prison for manslaughter. - Mother divorced father while he was incarcerated. - Father was moonshiner; exposed to alcohol at young age and doesn’t remember a time when he wasn’t drinking. - Began smoking at age 11; smoked ever since. - Father was emotionally and physically abusive to him and mother; he tried to take most of abuse to protect siblings and mother. Regularly witnessed father hitting mother until father was incarcerated. Beatings were worse when father was drunk. |

GUIDELINES FOR VERBAL FEEDBACK:

- Should be given to the learner from the ***patient perspective!***
- Should always include a behavioral example: “When you said x, it made me feel y.”
- Do not comment on medical information!
