# Supplementary material for: Communication Skills Training Using Simulation for Integrating Adverse Childhood Experiences into Adult Primary Care Visits for Students
Source: MedEdPORTAL. 2026 Nov 3;22:11648. doi: 10.15766/mep_2374-8265.11648 (PMC13728867; doi:10.15766/mep_2374-8265.11648)
Supplement: Supplementary file 1 [file mep_2374-8265.11648-s001.zip › F. Jonathan Jones Case.docx]

**DOOR NOTE**

**PATIENT/CLIENT INFORMATION:**

**NAME:** Jonathan Jones

**CHIEF COMPLAINT/REASON FOR VISIT:** Chronic pain, medication refill

**SETTING:** Ambulatory clinic, scheduled visit

**PERTINENT INFORMATION PROVIDED DURING CLINIC ORIENTATION:**

- You have been Jonathan’s PCP for several years and have seen him several times over the last year. You referred him to a pain management clinic 3 months ago for continued management of his chronic neck, back, and knee pain. He has been on narcotic pain medication to control his pain for over 10 years.
- Jonathan has tried physical therapy in the past but did not keep up with at-home exercises. He admits that movement does help with his low back pain.
- He also struggles with sleep apnea, chronic insomnia, hypertension, hypercholesterolemia, anxiety, depression, and type 2 diabetes.
- You have never discussed Jonathan’s childhood history with him.

**INSTRUCTIONS:**

Take a focused history and counsel patient on treatment plan. You can address the back/neck/knee pain briefly but your goal is to do a trauma inquiry with pt and if positive, discuss how that might be affecting his current health. If time permits, you might begin discussing treatment options for patients with history of trauma.

**ATTACHMENTS:**

**Facilitators:**

Print door notes for each room, observer schedule for each station.

Print RWJF ACEs infographic, attach to clipboards.

Print door notes.

**STANDARDIZED PATIENT TRAINING MATERIALS**

| **OVERVIEW** | **Event title:** Chronic disease and ACEs  **Event objectives for learner:**   1. Briefly address pt’s chief concern 2. Perform sensitive trauma inquiry including how pt feels trauma has affected health. Allow pt to determine extent of disclosure 3. Educate pt on how trauma affects the brain and body 4. Make a plan with patient for next steps   **Event format:** Individual, in-person encounter |
| --- | --- |
|  | **Case author:** M. Jelley, F. Wen, K. Coon, J. Miller-Cribbs, K. Foulks  **Date developed:** 2015  **Date revised:** 2026 |
| Affect or physical presentation: | Fatigued, experiencing tight pain in neck (cervical spine) and sharp pain in both knees. Rubs neck and knees every so often to indicate pain. |
| Clothing/moulage: | Clean, wearing clean street clothes. |
| Patient name & pronouns: | Jonathan Jones (he, him) |
| Sex and age range: | Male, 40-50 years |
| Chief complaint: | Pain assessment, difficulty sleeping, medication refill |
| Chief concern: | Unable to keep a job due to pain—may want help with application for disability. |
| Opening statement: | “My pain is really bothering me every day. I need a pain med refill and maybe disability.” |

**HISTORY OF THE PRESENT ILLNESS (HPI) TIMELINE**

Please refer to questioning & affect guidelines for instructions on delivery of this information.

| **Age 22** | Medically discharged from Air Force for service-related injuries. |
| --- | --- |
| **Last year** | MRI of spine showed mild disc bulging at lumbar spine and mild degenerative joint disease at lumbar and cervical spine. |
| **3 mos. ago** | Began seeing pain management; were providing narcotic prescription for chronic pain management. |
| **Last month** | Stopped seeing pain management; wanted him to start receiving steroid injections in his spine which he didn’t want. |

**QUESTIONING GUIDELINES**

| Information ***freely given*** if asked open-ended question(s): | **Qs about Reason for Visit:**  **Chronic Pain:** You ask for a refill of your narcotic medication for chronic low back and knee pain. The other, non-narcotic medications aren’t helping. You ask the doctor if you should go on disability. Your lower back feels tight (unable to bend over to pick things up from floor, rotate at waist, lift objects heavier than 5 pounds, walking increases pain, if asked) and both knees give sharp pains (unable to squat, bend at knees, walking increases pain, if asked). You’ve stopped seeing pain management because they want you to get spinal steroid injections which you don’t want.  **Qs about ACEs and Trauma History:** Your mother and father fought a lot and your dad used to beat your mom.  **If learner asks if anything has changed since your last visit or current stressors:** You are having more troubles at home because you haven’t been able to work in months and the bills are piling up. The only jobs you’ve been able to get require more manual or physical stamina (e.g., fast food) than you can manage with your pain.  **Qs about Efforts on Your Own:** “The only thing that works for my pain are the narcotic medications (oxycodone).” |
| --- | --- |
| Information provided ***only when learner asks specific question(s)***: | **Qs about Reason for Visit:**   1. **Chronic Pain:** Pain is worse with standing, lifting, bending, and walking. You can’t get comfortable to sleep more than a few hours at a time and you get tired easily. Pain and fatigue keep you from helping around the house. You previously tried PT which helped but stopped home exercises a while back. You admit that when you can get up and moving that movement helps some with the pain especially in your back. 2. **Family & Social Issues:** Issues began about 10 years ago during the recession when you were laid off from a well-paying job at a company that went bankrupt. There were few jobs in your field locally, and the lower paying jobs you had didn’t work out because of how much time you’d take off from work because of pain. Marriage is strained because wife is the bread winner and you and wife are worried about paying for college for your son and daughter (ages 17 and 15). You are not getting along with your wife or kids.    a. **If asked compassionately about what is going on in your relationships with your family…**”They all yell at me a lot for not being able to help around the house or work, and how it affects them and keeps them from what they want to do. I just go into my room and lie down in bed”. 3. **If there is a depression screen:** your mood is blue/down, and you are tired all the time, have difficulties concentrating, have memory problems, feel guilty. You spend your day trying to sleep because you sleep poorly at night (problems falling and staying asleep).   **Qs about ACEs and Trauma History:**   1. Your dad was put in prison for armed robbery and your mom then got a divorce and moved away    a. **If asked compassionately about your mom being treated violently,** you would stay in your bed with the covers over your head. 2. Your mom then raised you as a single parent and had a hard time supporting you both.    a. **If asked compassionately about what it was like** You admit that you sometimes didn’t have enough to eat, you didn’t have the right clothes to wear and got into fights a lot (if the student is really compassionate, you could also admit this was bullying). 3. You’re not sure but you think your mom was really depressed. She worked a lot and didn’t talk much to you, but you recall her yelling at you about certain things that could make your dad mad (making a mess, waking him up). 4. You were in Operational Support in the Air Force and had a non-combat related back injury during the service.    a. **If asked compassionately about what it was like** You admit that ops support is highly stressful with long hours, quick turnaround, difficult labor, bad communication, and high blame/responsibility when something is wrong with a plane.   **Link Between ACEs and Trauma History and current health or functioning (*this could be a question asking you if you are aware of a link, or it could be the learner providing a statement that there is a link and then using a metaphor or biological explanati*on)**   1. You have initial difficulty understanding the link to your chronic pain and/or depression: “I don’t see how this affects what I’m going through now.”    a. **…if the learner provides another attempt at an explanation** **but it is hard to understand or not delivered in empathic or supportive way** “I had not thought of that before, but even if I did, the past is in the past, so I am not sure it matters now.”    b. **…if the learner provides another attempt at an explanation** **and it is delivered in empathic or supportive way and/or is understandable** express that you can see how there could be a link for depression (if the student identifies this) and for your pain and ask “what will help me now with my pain and being depressed?” and then be open to suggestions for your treatment. |
| Information that ***must be provided***, even if not elicited: | When asked about chronic low back and knee pain, say “Can you help me get a refill of my medications and disability because I can’t work and I need money for me and my family?“ |
| Any **challenging questions** that patient should ask: | If the learner attempts a link between your ACEs and trauma with your chronic low back and knee pain that does not make sense to you, say “are you saying that my pain isn’t real? That it doesn’t come from being injured?” |
| SP response if no attempt to connect ACEs/Trauma to health is provided: | Focus on opioid medication and disability as the solutions to your concerns about your chronic low back and knee pain. |

**AFFECT GUIDELINES**

Affect changes throughout encounter based on dependent conversational variables.

| **If learner…** | **…then patient** |
| --- | --- |
| **Demonstrates active listening:** reflection of your concerns, validating feelings and experience, etc.  “I can understand”  “Thank you for repeating all of that”  “How has the medication helped you?”  “It sounds like…”  **No or poor active listening:** does not reflect concerns, validate feelings and experience  No verbal/nonverbal expression of empathy, but instead asks another question or directs conversation to another topic | Becomes visually less downcast, less flat in affect. Make better eye contact. Become more open to answering questions. Share the severity of your problems more readily.  Become more downcast, irritable, and distant. Cross your arms; uncross your arms and tap your fingers on your knee or arm of the chair. Occasionally give a hard stare at the student. Provide brief, unelaborated answers. Insist that the doctor focus on your request for help with your pain with medication and disability. |
| **Partners with patient:**  “What do you think about…”  “Do you have any ideas?”  “How can I help you?”  “What do you want?”  “Let’s solve this together?” | Respond in kind by being willing to problem solve.  “Well, PT did help before, but can it help me get to where I can work?”  “What else can I do to get back to work and get some income for my family?”  “I feel like I’ve just always had to ignore my feelings about things, and I’d like to feel different.” |
| **Gives information to patient about ACEs, neurobiological stress, link to physical and mental health conditions in adulthood:**  “Research shows that there is an association between difficult experiences in childhood and health problems in adulthood like depression and chronic pain…”  “ACEs are like a loaded backpack…  “Sometimes stress becomes so much it becomes toxic to us…”  “We learned certain ways of coping with feelings in childhood. But we can learn new ways of coping an rewire our brains. This treatment can help with that” | Be more physically open and curious about the discussion and trusting of the information provided. |
| **Prescriptive with patient:**  “You need to…”  “I want you to…” | Argue, resist, question, cross arms.  “Yes, but….”  “I’ve tried that and it doesn’t work” |
| **If learner is considerate, shows active listening, and makes an attempt (however successful) at linking ACEs and trauma to current health and functioning:** | Regardless of whether learner provided understandable information about ACEs and adult health outcomes, contribute to the discussion on developing a plan.  You ultimately will agree to treatment for your low back and knee pain, e.g., PT and NSAID pain medications.  If the learner has been empathic and considerate, you agree to counseling and medication for your depression and other plans to address social needs, e.g., vocational rehabilitation, family therapy. |

**PATIENT’S PAST MEDICAL HISTORY**

| General health: | Fair |
| --- | --- |
| Childhood illnesses: | N/A |
| Adult illnesses: | Sleep apnea, degenerative joint disease, chronic pain, chronic insomnia, type 2 DM, hypertension, hypercholesterolemia, anxiety, depression |
| Hospitalizations: | None (including mental health hospitalizations) |
| Surgical procedures: | **Procedure** \| **Location** \| **Date (year)**; N/A \| N/A \| N/A |
| Injuries/traumas: | N/A |
| Allergies/drug reactions: | **Allergen** \| **Reaction**; N/A \| N/A |
| Current medications: | **Name** \| **Treatment use** \| **OTC/Prescription** \| **Dosage**; Oxycodone \| Pain \| Prescription \| 10 mg q6 prn; **Medications for HTN, DM, hypercholesterolemia, etc. - irrelevant** \| \| \| |
| Family medical history: | N/A |

**SOCIAL HISTORY**

| Patient description | Marital status: \| Married; If married, how many years? \| Since 21; Children: *(Number & ages, if relevant)* \| 2, boy 17, girl 15; Occupation: \| Unemployed |
| --- | --- |
| Lifestyle/behavioral risks:  *(Include amount and duration)* | Stress: \| **Financial stressors**—currently unemployed, wife works. You lost lucrative job several years ago due to company relocation; found re-employment for much less pay in jobs requiring more manual effort. Can’t keep these jobs due to pain. **Family stressors**—Son planning on college in the fall; unsure of how to pay for college, causing more stress about finances. Daughter fallen in with wrong crowd, grades have dropped.; Tobacco: \| Non-smoker; Alcohol: \| None; Street drugs: \| None |
| Childhood trauma history: | - Father suffered from depression and regularly beat his mother in front of him. - Father was eventually incarcerated for armed robbery when he was 12. - Mother divorced father while he was incarcerated. |

GUIDELINES FOR VERBAL FEEDBACK:

- Should be given to the learner from the ***patient perspective!***
- Should always include a behavioral example: “When you said x, it made me feel y.”
- Do not comment on medical information!
