# Supplementary material for: Communication Skills Training Using Simulation for Integrating Adverse Childhood Experiences into Adult Primary Care Visits for Students
Source: MedEdPORTAL. 2026 Nov 3;22:11648. doi: 10.15766/mep_2374-8265.11648 (PMC13728867; doi:10.15766/mep_2374-8265.11648)
Supplement: Supplementary file 1 [file mep_2374-8265.11648-s001.zip › G. Janet Howard Case.docx]

**DOOR NOTE**

**PATIENT/CLIENT INFORMATION:**

**NAME:** Janet Howard

**CHIEF COMPLAINT/REASON FOR VISIT:** Knee pain, depression

**SETTING:** Ambulatory clinic, scheduled visit

**PERTINENT INFORMATION PROVIDED DURING CLINIC ORIENTATION:**

- You are the PCP for Janet and have seen her regularly for the past 3 years—ever since she moved to town.
- Janet has struggled with depressive symptoms for the past year but has maintained her sobriety from alcohol and drugs for over 8 years. She continues to struggle with tobacco abuse and generalized anxiety, for which she takes no medication nor does any therapy.
- You have never discussed any part of Janet’s childhood with her.

**INSTRUCTIONS:**

Take a focused history and counsel patient on treatment plan. You can address the knee pain briefly but your goal is to do a trauma inquiry with pt and if positive, discuss how that might be affecting her current health. If time permits, you might begin discussing treatment options for patients with history of trauma.

**ATTACHMENTS:**

**Facilitators:**

Print door notes for each room, observer schedule for each station.

Print RWJF ACEs infographic, attach to clipboards.

Print door notes.

**STANDARDIZED PATIENT TRAINING MATERIALS**

| **OVERVIEW** | **Event title:** Chronic disease and ACEs  **Event objectives for learner:**   1. Briefly address pt’s chief concern 2. Perform sensitive trauma inquiry including how pt feels trauma has affected health. Allow pt to determine extent of disclosure 3. Educate pt on how trauma affects the brain and body 4. Make a plan with patient for next steps   **Event format:** Individual, in-person encounter |
| --- | --- |
|  | **Case author:** M. Jelley, F. Wen, K. Coon, J. Miller-Cribbs, K. Foulks  **Date developed:** 2015  **Date revised:** 2026 |
| Required patient characteristics: | Normal weight, overweight, or obese |
| Affect or physical presentation: | Initially appear down, slightly sad, anxious, and guarded. At the beginning of the encounter your body language is closed-off (e.g., cross your arms, look away from learner).  As learner develops rapport with you, you may become more open to sharing current stressors and trauma history. See affect guidelines below. |
| Clothing/moulage: | Slightly less put together than a non-depressed person would be (little to no makeup, hair barely combed, wrinkled clothing, etc.) |
| Patient name & pronouns: | Janet Howard (she, her) |
| Sex and age range: | Female, 30-45 years |
| Chief complaint: | Knee pain, depression |
| Chief concern: | Getting on disability so you can stop having to go to work |
| Opening statement: | “My knee is really bothering me and makes it really hard to get in and out of the house. I just feel like it’s too much trouble and would rather just be able to stay in bed all day.” |

**HISTORY OF THE PRESENT ILLNESS (HPI) TIMELINE**

Please refer to questioning & affect guidelines for instructions on delivery of this information.

| **2 years ago** | Began having weight issues |
| --- | --- |
| **1 year ago** | Began experiencing depressed mood, crying spells, difficulties concentrating, loss of pleasure in activities, decreased energy, psychomotor retardation, low self-esteem, excessive guilt, and increased and associated weight gain. |
| **2 weeks ago** | Walking up the stairs to your apartment and felt a painful pop in your left knee. Non-radiating pain is worse with standing and walking, especially up and down stairs or inclines. Associated symptoms include swelling, clicking, popping, weakness, and instability. |
| **Today** | Experiencing all symptoms listed above. |

**QUESTIONING GUIDELINES**

| Information ***freely given*** if asked open-ended question(s): | **Qs about Reason for Visit:**   1. **Depression:** Been feeling down for a while, but is worse lately and you are having trouble with oversleeping, you are crying a lot and eating a lot, feeling that life is horrible and you’re a terrible person. 2. **Knee Pain:** Chronic knee pain has been bad since 2 weeks ago when you were walking up the stairs to your apartment and the knee gave out. You ask the doctor if you can go on disability.   **Qs about ACEs and Trauma History:** Your mother died when you were little and your dad married and divorced a few times before you were 21. You ended up being the mom to your little sister. You partied a lot and got into some not good situations, but you’ve been sober now for the past 8 years.  **If learner asks if anything has changed since your last visit or current stressors:** You can’t manage anymore at work because of worse knee pain, and because you can’t focus or seem to have the energy to get through the day. Your life overall “stinks” or “sucks” because you moved to be close to your sister who now doesn’t seem to want to spend time with you**.**  **Qs about Efforts on Your Own:** You keep off your knee, elevate and ice it as much as possible which provides some, temporary relief.  “I don’t know what to do about anything else. I can’t get my sister to respond to my texts or calls, but this has always the ways she’s been – it’s only on her terms.  “I eat my feelings, but I’m not using drugs or alcohol!”. |
| --- | --- |
| Information provided ***only when learner asks specific question(s)***: | **Qs about Reason for Visit:**   1. **Depression** Been feeling a little blue for a while, but is worse lately and you are now feeling really down, and are having trouble with oversleeping and over eating at meals and on unhealthy snacks. You sometimes start crying for no reason, and have recently begun feeling overwhelmed by your stressful job. You are worried about your weight gain because you have lost weight over the past 2 years through reducing sweets food/drinks and changing to healthy snacks. Not suicidal/homicidal. 2. **Knee Pain** Pain is worse with standing and walking. Your knee feels swollen, and now clicks and pops, as well as feels unstable and weak. You are insistent about wanting disability and the doctor’s help with this.   **Qs about ACEs and Trauma History:**   1. **Mom’s death** – She died in a car accident when you were 4 y.o.    a. **If asked about how this affected you**: Your dad dated a lot and had 3 marriages, and he also moved you and your sister a lot.    b. **If asked about how dad was doing**: He was an alcoholic and always out of the house at bars to meet women which left you to look after your little sister 2. **Childhood sexual abuse** – You were raped at age 14 by an uncle. You didn’t go for therapy or counseling because your “dad was too busy chasing women.” You don’t want to talk anymore about it to the learner.   **Link Between ACEs and Trauma History and current health or functioning (*this could be a question asking you if you are aware of a link, or it could be the learner providing a statement that there is a link and then using a metaphor or biological explanati*on)**   1. Agree with connection between your previous use of alcohol, marijuana and meth to cope with your childhood (and rape if you disclosed the rape) because “I did this work in AA”, but say “I don’t see how this affects my knee pain”    a. **…if the learner provides another attempt at an explanation** **but it is hard to understand or not delivered in empathic or supportive way “**OK, so you are saying that what happened in the past is affecting my physical health, my pain now. But I’m still not sure I get it”    b. **…if the learner provides another attempt at an explanation** **and it is delivered in empathic or supportive way and/or is understandable** express that you can see how there could be a link, and ask “what now” and then be open to suggestions for your treatment |
| Information that ***must be provided***, even if not elicited: | When asked about knee pain, say, “I think I really need disability so I can have time off work and so my knee can heal.” |
| Any **challenging questions** that patient should ask: | If the learner attempts a link between your ACEs and trauma with your knee pain that does not make sense to you, say “so you think the pain is all in my head?” |
| SP response if no attempt to connect ACEs/Trauma to health is provided: | Focus on disability as the solution to your concerns about your knee pain and depression. |

**AFFECT GUIDELINES**

Affect changes throughout encounter based on dependent conversational variables.

| **If learner…** | **…then patient** |
| --- | --- |
| **Demonstrates active listening:** reflection of your concerns, validating feelings and experience, etc.  “I can understand”  “Thank you for repeating all of that”  “What helps you cope?”  “It sounds like…”  **No or poor active listening:** does not reflect concerns, validate feelings and experience.  No verbal/nonverbal expression of empathy, but instead asks another question or directs conversation to another topic | Becomes visually less downcast, less flat in affect. Make better eye contact. Become more open to answering questions. Share the severity of your problems more readily.  Become more downcast and distant. Cross your arms. Do not make eye contact. Provide brief, unelaborated answers. Insist that the doctor focus on your request for disability. |
| **Partners with patient:**  “What do you think about…”  “Do you have any ideas?”  “How can I help you?”  “What do you want?”  “Let’s solve this together?” | Respond in kind by being willing to problem solve.  “Well, elevating my knee is all that’s been helping. What else can I do?”  “I am really tired of feeling like my life stinks/sucks, and I’d like to feel different”  “I feel like I’ve just always had to take care of everything by myself, and I don’t want to feel so alone anymore.” |
| **Gives information to patient about ACEs, neurobiological stress, link to physical and mental health conditions in adulthood:**  “Research shows that there is an association between difficult experiences in childhood and health problems in adulthood like depression and chronic pain…”  “ACEs are like a loaded backpack…  “Previous trauma can turn up the volume on pain”  “Sometimes stress becomes so much it becomes toxic to us…”  “This treatment can help with…” | Be more physically open and curious about the discussion and trusting of the information provided. |
| **Prescriptive with patient:**  “You need to…”  “I want you to…” | Argue, resist, question, cross arms.  “Yes, but….”  “I’ve tried that and it doesn’t work” |
| **If learner is considerate, shows active listening, and makes an attempt (however successful) at linking ACEs and trauma to current health and functioning:** | Regardless of whether learner provided understandable information about ACEs and adult health outcomes, contribute to the discussion on developing a plan.  You ultimately will agree to treatment for your knee pain, e.g., PT and continuation of your pain medications.  If the learner has been empathic and considerate, you agree to counseling and medication for your depression and other plans to address social isolation, e.g., identifying/joining social groups. |

**PATIENT’S PAST MEDICAL HISTORY**

| General health: | Fair |
| --- | --- |
| Childhood illnesses: | N/A |
| Adult illnesses: | Tobacco use, generalized anxiety |
| Hospitalizations: | None (including mental health hospitalizations) |
| Surgical procedures: | **Procedure** \| **Location** \| **Date (year)**; N/A \| N/A \| N/A |
| Injuries/traumas: | N/A |
| Allergies/drug reactions: | **Allergen** \| **Reaction**; N/A \| N/A |
| Current medications: | **Name** \| **Treatment use** \| **OTC/Prescription** \| **Dosage**; Irrelevant to case \| \| \| |
| Psychiatric history: | Thoughts of suicide: \| No; Thoughts of harming others: \| No; Mental health treatment: \| No formal treatment |
| Family medical history: | N/A |

**SOCIAL HISTORY**

| Patient description | Marital status: \| Never married; If married, how many years? \| N/A; Children: *(Number & ages, if relevant)* \| 0; Occupation: \| Tax accountant |
| --- | --- |
| Lifestyle/behavioral risks:  *(Include amount and duration)* | Eating habits: \| Over- eating when stressed; Exercise: \| None; Sleeping habits: \| N/A; Stress: \| - Strained relationship with only sister who lives in town. - Job (tax accountant) is very stressful—causing increased feelings of depression (depressed mood, crying spells, difficulties concentrating, loss of pleasure in activities, decreased energy, psychomotor retardation, low self-esteem, excessive guilt, increased appetite and associated weight gain). - In significant debt due to credit cards and student loans. - Moved to area 3 years ago to be near sister. - Recently ended a long-distance relationship and has very few friends in the area.; Tobacco: \| 1.5 ppd since age 15; Alcohol: \| Recovering from alcohol and drug abuse, sober for 8 years (AA program successful) and very proud of sobriety.; Street drugs: \| |
| Childhood trauma history: | - Mother died at age 4, father remarried and divorced 3 times - Father relocated frequently, little stability in childhood - Raped at age 14 by uncle, you don’t like to talk about it - Throughout teens to late 20’s, dependent on alcohol and marijuana, abused stimulants, had multiple sex partners and unsafe sex practices |

GUIDELINES FOR VERBAL FEEDBACK:

- Should be given to the learner from the ***patient perspective!***
- Should always include a behavioral example: “When you said x, it made me feel y.”
- Do not comment on medical information!
