# Supplementary material for: Communication Skills Training Using Simulation for Integrating Adverse Childhood Experiences into Adult Primary Care Visits for Students
Source: MedEdPORTAL. 2026 Nov 3;22:11648. doi: 10.15766/mep_2374-8265.11648 (PMC13728867; doi:10.15766/mep_2374-8265.11648)
Supplement: Supplementary file 1 [file mep_2374-8265.11648-s001.zip › H. Faculty Guide.docx]

**Faculty Guide: The Professional ACEs-Informed Training for Health (PATH)**

**Purpose**

This guide prepares faculty to facilitate the PATH simulation. Faculty do not need to be experts in adverse childhood experiences (ACEs); rather, they should understand the goals of trauma-informed communication and be comfortable facilitating reflective discussion. This curriculum has been implemented across multiple health professions, including medicine, nursing, physician assistant, occupational therapy, and social work.

**Faculty Philosophy**

The primary role of the faculty facilitator is to create a psychologically safe learning environment in which learners can practice trauma-informed communication with confidence. The goal of the simulation is not to achieve a perfect patient encounter, but to provide an opportunity for deliberate practice, reflection, and formative feedback. Faculty should approach the experience as coaches who guide learner growth rather than evaluators searching for errors. Multiple effective communication styles may be appropriate provided they reflect the principles of trauma-informed care and patient-centered communication. Throughout the simulation and debriefing, faculty should consistently reinforce the five PATH communication skills, introducing and explaining ACEs, demonstrating empathy, reducing stigma, and engaging patients in shared decision-making, as the foundation of trauma-informed clinical communication.

**Adaptation**

The training described involved use of paid simulated patients and simulation center time. As these resources are not available to some educators, there are adaptations that can be used. The facilitation strategies described in this guide are applicable regardless of simulation modality. The curriculum may be implemented using simulated patients, trained actors, peer role-play, faculty role-play, or video demonstrations when simulation center resources are unavailable. Faculty should maintain the same emphasis on communication practice, structured debriefing, and formative feedback regardless of format.

**Prior to the Session**

Faculty should review:

- Learning objectives
- Simulated patient cases (Appendices D-G)
- PATH communication model (incorporated into didactic slides, Appendix B)
- Clinical skills checklist
- Debriefing quick guide (Appendix I)
- Learner resources (Appendix A)

Recommended preparation time: **30–60 minutes**

**Logistics**

Faculty should arrive before learners review the cases, confirm simulated patient preparation, verify audiovisual equipment (if used), and review timing for each simulation encounter. Faculty should ensure that all participants understand expectations for confidentiality, respectful discussion, and psychological safety before beginning the activity.

**Educational Goals**

The primary goal of the simulation is to develop trauma-informed communication skills rather than evaluate factual knowledge about ACEs. Learners practice integrating trauma-informed communication into routine clinical encounters while demonstrating the five PATH communication skills. Faculty should coach learners to demonstrate the five PATH communication skills:

1. Introduce and connect ACEs to health
2. Explain ACEs using patient-centered language
3. Demonstrate empathy
4. Reduce stigma and normalize discussion
5. Engage in collaborative treatment planning and shared decision-making

**Faculty Role During Simulation**

Faculty primarily serve as coaches and observers.

Responsibilities include:

- Maintain a psychologically safe learning environment.
- Observe communication behaviors rather than factual recall.
- Allow learners to work through difficult moments before intervening.
- Reinforce effective communication first.
- Provide constructive feedback using the PATH framework.
- Encourage reflective learning rather than identifying a single "correct" response.

**FEEDBACK FOLLOWING SIMULATIONS**

**(see also Quick Guide for Post-Simulation Feedback Session)**

**Simulated Patient Feedback**

Simulated patients should provide feedback from the patient's perspective rather than evaluating clinical knowledge. Faculty should encourage SPs to describe how the learner's communication affected feelings of safety, trust, understanding, and engagement. Patient-centered feedback often reinforces aspects of empathy and stigma reduction that faculty may not directly observe. Faculty should invite SP feedback before providing their own observations, as patients often identify aspects of communication that faculty observers may not recognize.

**Giving Feedback on the Individual Simulation**

The feedback is the most important component of the learning experience and should occur immediately following each SP encounter. Faculty should approach the discussion as facilitators rather than lecturers. The purpose is to help learners reflect on their communication, clinical reasoning, and emotional responses rather than to identify a single "correct" way to conduct the encounter. Faculty should create a psychologically safe, respectful, and nonjudgmental environment in which learners feel comfortable discussing both strengths and challenges. Faculty should resist the temptation to immediately "correct" learner performance. Instead, allow learners to reflect on their own decision-making before offering faculty observations.

Faculty should remind learners that this is a safe place to practice skills, not a test or OSCE.

The PATH curriculum uses a three-phase model adapted from experiential learning principles:

**Phase 1: Reflection and Description**

Begin by asking learners to describe the encounter in their own words before offering faculty	observations.

Sample questions:

- Tell us what happened during your encounter.
- What do you think went well?
- What was most challenging?
- How did the patient respond to your approach?

This phase allows faculty to understand how learners interpreted the encounter and provides insight	into their clinical reasoning.

**Phase 2: Analysis and Personalization**

Guide learners in examining why they made particular communication choices and how those choices	influenced the interaction.

Sample questions:

- What were you thinking when you introduced ACEs?
- Why did you choose that response?
- What emotions were you noticing in yourself?
- How do you think the patient interpreted your response?
- What alternative approaches might have been effective?

Faculty should encourage participation from peer observers and SPs while maintaining a supportive atmosphere focused on learning rather than criticism.

**Phase 3: Generalization and Application**

Conclude by helping learners connect the simulation to future clinical practice.

Sample questions:

- How might you apply this experience with future patients?
- What communication skills will you continue to practice?
- How has this simulation influenced your thinking about trauma-informed care?
- What is your primary take-home message from today's experience?

The debrief should conclude with each learner identifying one specific communication strategy they	plan to incorporate into future patient encounters.

**Tips for Success**

Experiences with implementing PATH across multiple professions suggests several strategies that enhance learner engagement:

- Focus on communication rather than diagnosis.
- Encourage curiosity instead of perfection.
- Allow silence after emotionally difficult disclosures.
- Reinforce empathy before discussing treatment options.
- Connect feedback directly to the five PATH communication skills.
- Ask each learner to identify a personal "take-home message" at the end of the session.

**Common Pitfalls**

Avoid:

- Turning the encounter into an ACE knowledge quiz.
- Interrupting learners too quickly during feedback session.
- Correcting every missed opportunity.
- Spending excessive time discussing ACE epidemiology.
- Allowing students to mimic simulation demonstration, thus not using their own communication styles
- Focusing primarily on referrals before establishing rapport and patient buy-in (common student pitfall)
- Rushing or shortening the feedback because of time constraints.

**Using the Clinical Skills Rubric**

The clinical skills rubric is designed to evaluate observable communication behaviors rather than factual knowledge about ACEs. Faculty can complete the rubric immediately following each encounter while the interaction is fresh. The rubric is intended to support formative feedback and guide learner development rather than to assign a pass/fail judgment. Faculty should approach the scoring process as coaches who facilitate learner growth rather than evaluators searching for errors.

Faculty should evaluate learner performance across the five core PATH communication skills:

- Introduces and connects ACEs to current health concerns in a respectful, patient-centered manner.
- Explains the relationship between childhood adversity and health using language that is understandable and appropriate for the patient.
- Demonstrates empathy through verbal and nonverbal communication, active listening, and validation of the patient's experiences.
- Reduces stigma by normalizing discussion of adversity and conveying acceptance without judgment.
- Engages the patient in shared decision-making by collaboratively discussing next steps and treatment options.

The rubric is behaviorally anchored and designed to assess communication behaviors rather than overall clinical competence. Faculty should score only behaviors that were directly observed during the encounter and avoid inferring learner intentions. Ratings should reflect the learner's developmental stage and expected level of training rather than comparison with expert performance. Multiple communication styles may demonstrate competency provided they are consistent with trauma-informed care principles.

When completing the rubric, faculty should:

- Evaluate behaviors that were directly observed.
- Consider the learner's developmental level and expected level of proficiency.
- Reinforce strengths before discussing opportunities for improvement.
- Use specific examples from the encounter to support feedback.
- Encourage learner self-assessment before sharing faculty observations.

Communication skills develop over time, and learners often demonstrate strengths in some PATH domains while requiring additional coaching in others. Faculty should consider the five communication skills together rather than focusing on individual checklist items. For example, a learner may effectively introduce ACEs but require additional coaching in demonstrating empathy or engaging the patient in shared decision-making. The overall pattern of performance should guide individualized coaching and reflection.

**Faculty Calibration**

When multiple faculty facilitators are involved, a brief calibration session is recommended before the simulation. Faculty can independently score a sample video encounter using the clinical skills rubric and then compare ratings to establish shared expectations for learner performance and promote consistency in scoring and formative feedback.

Calibration discussions should emphasize that the purpose of the rubric is to support learner development rather than identify errors. The goal of the simulation is not to produce a perfect encounter, but to provide a safe environment in which learners practice difficult conversations, receive constructive feedback, and build confidence in trauma-informed communication.

**Psychological Safety and Learner Well-Being**

Discussion of childhood adversity may evoke personal experiences.

Faculty should:

- Normalize emotional responses.
- Create a respectful, psychologically safe learning environment.
- Encourage learners to access institutional wellness or counseling resources if needed.
- Briefly address self-care and secondary traumatic stress as part of the learning process

**Pre-Simulation Faculty Checklist**

☐ Review learning objectives

☐ Review patient case

☐ Review PATH communication skills

☐ Review simulated patient guidance

☐ Review evaluation rubric

☐ Review debriefing guide

☐ Confirm simulation logistics

☐ Review training resources

**Structured faculty guide for large group debrief following simulations**

**Questions to ask:**

1. How do you all feel about the simulation?
2. Can you share one thing you learned from this experience?
3. What do you think went well?
4. Can you share with us what you found to be the most challenging part of the simulation?
5. If you were to do this again, what would you change?
6. How could the educational experience be improved?
7. Can you share one skill learned from this simulation you plan on implementing in your practice?

**Supporting Materials**

**Appendix A. ACEs Training Resources**

**Appendices D-G. Simulated Patient Training Guide**

**Appendix I. Quick Guide for Post-Simulation Feedback**

**Appendix L. Clinical Skills Rubric and Checklist**
