# Supplementary material for: Communication Skills Training Using Simulation for Integrating Adverse Childhood Experiences into Adult Primary Care Visits for Students
Source: MedEdPORTAL. 2026 Nov 3;22:11648. doi: 10.15766/mep_2374-8265.11648 (PMC13728867; doi:10.15766/mep_2374-8265.11648)
Supplement: Supplementary file 1 [file mep_2374-8265.11648-s001.zip › I. Quick Guide for Postsimulation Feedback.docx]

**Quick Guide for Postsimulation Feedback Session**

| **Questions from SP/faculty to facilitate feedback:** | **Questions to SP to provide learner direct feedback on specific skills:** |
| --- | --- |
| How do you think that went? | How did you feel about the introduction of ACEs to the conversation? <If they need more -> Did it feel natural? Did it feel rushed? Did the timing make sense to you? |
| Is there anything you think went particularly well or you felt really good about? | Did you understand the concept of ACEs when the learner explained it? |
| Is there anything you would go back and change? | Did you understand how ACEs can impact your health, even as an adult? |
| Was there any part of the encounter that surprised you? <If they need more -> Were there any pleasant surprises? Maybe not so pleasant surprises? | Did the learner demonstrate empathy when you were telling your story (if you gave them an opportunity)? |
| Was anything easier than you anticipated?  Was anything more challenging that you anticipated? | Did you feel heard and understood? |
|  | Did the learner attempt to normalize the experience of ACEs? |
|  | Did the learner ask you about your goals for this visit? |
|  | Did the learner engage you in your treatment planning? |
|  | Did the learner partner with you to make a plan for your future visits? |
