# Supplementary material for: Communication Skills Training Using Simulation for Integrating Adverse Childhood Experiences into Adult Primary Care Visits for Students
Source: MedEdPORTAL. 2026 Nov 3;22:11648. doi: 10.15766/mep_2374-8265.11648 (PMC13728867; doi:10.15766/mep_2374-8265.11648)
Supplement: Supplementary file 1 [file mep_2374-8265.11648-s001.zip › J. ACEs Training Agenda.docx]

ACEs Simulation Training – Agenda example for half day session

| **Time** | **Topic** | **Facilitator(s)** |
| --- | --- | --- |
| 1:00 – 1:10 pm  10 minutes  **Location** | Participant check-in and completion of pre-evaluation | Facilitator Names |
| 1:10 –1:50 pm  40 minutes  **Location** | **Adverse Childhood Experiences (ACEs) lecture:** Introduction to ACEs, TIC, and PATH Model | Facilitator Names |
| 1:50 – 2:20 | **Preparation for Simulation:** case demonstration and cases discussion |  |
| 2:20 – 2:30 pm  30 minutes  **Location** | Move to sim center/ break | Facilitator Names |
| 2:30 - 3:40 pm  80 minutes  **Location** | **Outpatient Visit Simulation (Sim Center Clinic Rooms):**  10 minute visit followed by 5-10 minute debrief with faculty and SP  6 SP rooms with 6 faculty – 4 rounds of simulation (24 total students)* | Facilitator Names |
| 3:40 - 3:50 pm  10 minutes | Short break |  |
| 3:50 - 4:15 pm  20 minutes  **Location** | Group Debrief (see guide) + Completion of post-Evaluation | Facilitator Names |

*Varies depending on the number of learners, SPs, and rooms.
