# Supplementary material for: Communication Skills Training Using Simulation for Integrating Adverse Childhood Experiences into Adult Primary Care Visits for Students
Source: MedEdPORTAL. 2026 Nov 3;22:11648. doi: 10.15766/mep_2374-8265.11648 (PMC13728867; doi:10.15766/mep_2374-8265.11648)
Supplement: Supplementary file 1 [file mep_2374-8265.11648-s001.zip › K. Pre- and Posttraining Survey.docx]

**Pre-training ACEs training survey**

*Note: Students completed this survey in Qualtrics.*

1. Rate your general knowledge on Adverse Childhood Experiences

No knowledge

A little bit of knowledge

A moderate amount of knowledge

A lot of knowledge

2. Rate your general knowledge on the link between traumatic exposures and poor health outcomes

No knowledge

A little bit of knowledge

A moderate amount of knowledge

A lot of knowledge

3. Rate your general knowledge on the principles of trauma-informed care

No knowledge

A little bit of knowledge

A moderate amount of knowledge

A lot of knowledge

4. Rate your general knowledge on resources to support patients with a history of traumatic exposures

No knowledge

A little bit of knowledge

A moderate amount of knowledge

A lot of knowledge

5. Rate how important you feel it is to ask about traumatic exposures during visits in primary care

Not at all important

Slightly important

Moderately important

Very important

6. Rate how important you feel it is to discuss the relationship between trauma exposure and poor health outcomes with patients

Not at all important

Slightly important

Moderately important

Very important

7. Please rate how important you believe it is to have a discussion of patient strengths and resiliency during routine visits in primary care

Not at all important

Slightly important

Moderately important

Very important

8. Rate your confidence with incorporating the principles of trauma-informed care into your patient visits

Not at all confident

Slightly confident

Moderately confident

Very confident

9. Rate your confidence in inquiring about traumatic exposures into your patient visits

Not at all confident

Slightly confident

Moderately confident

Very confident

10. Rate your confidence in incorporating discussion with patients regarding the relationship between history of traumatic exposures and poor health outcomes into your patient visits

Not at all confident

Slightly confident

Moderately confident

Very confident

11. Rate your confidence in incorporating discussion of resiliency/strengths-building into your routine patient visits

Not at all confident

Slightly confident

Moderately confident

Very confident

12. Rate your confidence in responding to a patient disclosure of a traumatic past with a trauma-informed approach

Not at all confident

Slightly confident

Moderately confident

Very confident

13. Rate your confidence in offering resources of support to a patient who discloses a history of a trauma

Not at all confident

Slightly confident

Moderately confident

Very confident

**Post-training ACEs training survey**

*Note: Students completed this survey in Qualtrics.*

1. Rate your general knowledge on Adverse Childhood Experiences

No knowledge

A little bit of knowledge

A moderate amount of knowledge

A lot of knowledge

2. Rate your general knowledge on the link between traumatic exposures and poor health outcomes

No knowledge

A little bit of knowledge

A moderate amount of knowledge

A lot of knowledge

3. Rate your general knowledge on the principles of trauma-informed care

No knowledge

A little bit of knowledge

A moderate amount of knowledge

A lot of knowledge

4. Rate your general knowledge on resources to support patients with a history of traumatic exposures

No knowledge

A little bit of knowledge

A moderate amount of knowledge

A lot of knowledge

5. Rate how important you feel it is to ask about traumatic exposures during visits in primary care

Not at all important

Slightly important

Moderately important

Very important

6. Rate how important you feel it is to discuss the relationship between trauma exposure and poor health outcomes with patients

Not at all important

Slightly important

Moderately important

Very important

7. Please rate how important you believe it is to have a discussion of patient strengths and resiliency during routine visits in primary care

Not at all important

Slightly important

Moderately important

Very important

8. Rate your confidence with incorporating the principles of trauma-informed care into your patient visits

Not at all confident

Slightly confident

Moderately confident

Very confident

9. Rate your confidence in inquiring about traumatic exposures into your patient visits

Not at all confident

Slightly confident

Moderately confident

Very confident

10. Rate your confidence in incorporating discussion with patients regarding the relationship between history of traumatic exposures and poor health outcomes into your patient visits

Not at all confident

Slightly confident

Moderately confident

Very confident

11. Rate your confidence in incorporating discussion of resiliency/strengths-building into your routine patient visits

Not at all confident

Slightly confident

Moderately confident

Very confident

12. Rate your confidence in responding to a patient disclosure of a traumatic past with a trauma-informed approach

Not at all confident

Slightly confident

Moderately confident

Very confident

13. Rate your confidence in offering resources of support to a patient who discloses a history of a trauma

Not at all confident

Slightly confident

Moderately confident

Very confident

14. Please tell us what you liked and did not like or any suggested improvements for our ACEs/Trauma-Informed Training.
