# Supplementary material for: Communication Skills Training Using Simulation for Integrating Adverse Childhood Experiences into Adult Primary Care Visits for Students
Source: MedEdPORTAL. 2026 Nov 3;22:11648. doi: 10.15766/mep_2374-8265.11648 (PMC13728867; doi:10.15766/mep_2374-8265.11648)
Supplement: Supplementary file 1 [file mep_2374-8265.11648-s001.zip › L. Clinical Skills Rubric and Checklist.docx]

| **Rubric for Resident Evaluation** | | | | | |
| --- | --- | --- | --- | --- | --- |
| **Goal One: Introduction of ACEs into Encounter** | | | | | |
| 1  Unsatisfactory | 2  Marginal | 3  Competent | 4  Excellent | 5  Exceptional | |
| ACEs not mentioned or ineffectively introduced |  | ACEs introduced; seems forced into the encounter or disjointed (overly directed by the provider; did not flow naturally from patient’s presenting problems/issues) |  | ACEs naturally and seamlessly woven into the flow of the encounter (exceptional; grounded in information from the patient’s history) | |
| **Goal Two: Explanation of Link between ACEs and Current Health Status** | | | | | |
| 1  Unsatisfactory | 2  Marginal | 3  Competent | 4  Excellent | 5  Exceptional | |
| Link between ACEs and current health issues not established |  | Elementary link of ACEs to current health issues; utilized jargon (used much medical/professional jargon, not lay language; did not consider health literacy of the patient) |  | Skillfully linked ACEs with current health issues (excellent job linking ACEs and current health status; did not use medical/professional jargon; used handouts/metaphors) | |
| **Goal Three: Expressions of Accurate Empathy** | | | | | |
| 1  Unsatisfactory | 2  Marginal | 3  Competent | 4  Excellent | 5  Exceptional | |
| No empathic expressions or judgmental, dismissive |  | Some empathic expressions; words and body language match to communicate empathy |  | Multiple expressions of accurate verbal and non-verbal empathy (expressions always on point, permeated tone/ tenor of interaction) | |
| **Goal Four: Reduction of ACEs Stigma (focus on reducing isolation, shame, blame)** | | | | | |
| 1  Unsatisfactory | 2  Marginal | 3  Competent | 4  Excellent | 5  Exceptional | |
| Did not address potential ACEs stigma or discouraged patient disclosure (uncomfortable trauma discussion) |  | Addressed potential ACEs stigma (used some stigma-reducing statements such as, “you’re not alone”) |  | Successfully addressed potential ACEs stigma, very effective (used statements to reduce isolation, shame, and blame) | |
| **Goal Five: Collaborative Decision Making** | | | | | |
| 1  Unsatisfactory | 2  Marginal | 3  Competent | 4  Excellent | 5  Exceptional | |
| Did not seek patient’s input into treatment formulation plan |  | Sought patient’s input into treatment formulation, provider-directed plan developed |  | Actively sought patient’s input into treatment formulation, collaborative plan developed | |
|  | | | | |  |
| **Overall Performance** | | | | | |
| 1  Unsatisfactory | 2  Marginal | 3  Competent | 4  Excellent | 5  Exceptional | |
| Struggled to meet any of the goals of the encounter |  | Attempted most goals of the encounter |  | Exemplary implementation of goals of the encounter | |
| **Level of Simulated Patient Difficulty** | | | | | |
| **Patient Agreeableness** | | | | | |
| 1 | 2 | 3 | 4 | 5 | |
| Strongly opposed to connecting ACEs to current health problems; unable to consider a multimodal treatment plan |  | Resistant to connecting ACEs with current health issues; hesitantly agrees with a multimodal treatment plan |  | Quickly accepts connecting ACEs with current health issues; very agreeable to a multimodal treatment plan | |
| **Patient Reactivity** | | | | | |
| 1 | 2 | 3 | 4 | 5 | |
| Loss of composure, exhibits strong, negative affect during ACEs discussion Pleasant and non-emotionally reactive during discussion of ACEs |  | Maintains overall composure, display of upset during discussion of ACEs |  | Pleasant and non-emotionally reactive during discussion of ACEs | |

*.*
